# *Blastocystis* Mitochondrial Genomes Appear to Show Multiple Independent Gains and Losses of Start and Stop Codons

**DOI:** 10.1093/gbe/evw255

**Published:** 2016-11-09

**Authors:** Alison S. Jacob, Lee O’Brien Andersen, Paulina Pavinski Bitar, Vincent P. Richards, Sarah Shah, Michael J. Stanhope, C. Rune Stensvold, C. Graham Clark

**Affiliations:** ^1^Faculty of Infectious and Tropical Diseases, London School of Hygiene and Tropical Medicine, London, United Kingdom; ^2^Present address: Faculty of Natural Sciences, Imperial College, London, United Kingdom; ^3^Department of Microbiology and Infection Control, Statens Serum Institut, Copenhagen, Denmark; ^4^Department of Population Medicine and Diagnostic Sciences, Cornell College of Veterinary Medicine, Cornell University, Ithaca, NY; ^5^Present address: Department of Biological Sciences, College of Agriculture, Forestry and Life Sciences, Clemson University, Clemson, SC; ^6^Department of Biochemistry and Molecular Biology, Dalhousie University, Halifax, Nova Scotia, Canada

**Keywords:** mitochondrion, phylogeny, stramenopile, tRNA

## Abstract

Complete mitochondrion-related organelle (MRO) genomes of several subtypes (STs) of the unicellular stramenopile *Blastocystis* are presented. Complete conservation of gene content and synteny in gene order is observed across all MRO genomes, comprising 27 protein coding genes, 2 ribosomal RNA genes, and 16 transfer RNA (tRNA) genes. Despite the synteny, differences in the degree of overlap between genes were observed between subtypes and also between isolates within the same subtype. Other notable features include unusual base-pairing mismatches in the predicted secondary structures of some tRNAs. Intriguingly, the *rps4* gene in some MRO genomes is missing a start codon and, based on phylogenetic relationships among STs, this loss has happened twice independently. One unidentified open reading frame (*orf160*) is present in all MRO genomes. However, with the exception of ST4 where the feature has been lost secondarily, *orf160* contains variously one or two in-frame stop codons. The overall evidence suggests that both the *orf160* and *rps4* genes are functional in all STs, but how they are expressed remains unclear.

## Introduction

Mitochondria exist in many divergent forms, particularly in eukaryotes that inhabit anaerobic or low oxygen environments (Hjort et al. 2010; Lithgow and Schneider 2010; Müller et al. 2012; Makiuchi and Nozaki 2014). The organelles of such eukaryotes differ substantially from the mitochondria of textbooks in genome content and biochemical pathways, with the former often exhibiting much reduced gene repertoires.

One example of such mitochondrion-related organelles are hydrogenosomes, which produce hydrogen and ATP via substrate level phosphorylation (Lindmark and Müller 1973; Müller 1993) and, with one known exception (de Graaf et al. 2011), lack an organellar genome (Makiuchi and Nozaki 2014). There are also mitosomes (Tovar et al. 1999), which are organelles that have completely lost a genome and the ability to produce ATP but retain essential aspects of sulphur metabolism (Ali and Nozaki 2013). Organelles also exist that have characteristics intermediate between those of textbook mitochondria and hydrogenosomes (Stechmann et al. 2008); these organelles are often termed mitochondrion-like organelles (MLOs) or mitochondrion-related organelles (MROs). One example of an MRO is that found in the *Blastocystis*, which exhibits a membrane potential, contains an organellar genome (Nasirudeen and Tan 2004), and is involved in ATP production (Hamblin et al. 2008), but although the MRO contains a hydrogenase protein the production of hydrogen has not been confirmed. The *Blastocystis* MRO harbors a complex suite of metabolic processes (Stechmann et al. 2008; Denoeud et al. 2011) and is perhaps better termed an anaerobic mitochondrion.

*Blastocystis* is a member of the stramenopiles (also known as Heterokonts) and is an ubiquitous constituent of the intestinal microflora of mammalian, avian, reptilian, and arthropod hosts (Clark et al. 2013). Phylogenetic reconstructions based on small subunit ribosomal RNA gene (SSU rDNA) sequences have shown that the genus is clearly demarcated into 17 clades, termed subtypes (STs), in mammals and birds alone (Alfellani et al. 2013), and that genetic divergence of this gene within subtypes can be as high as 3% (Stensvold et al. 2007, 2012). These genetically diverse organisms are morphologically indistinguishable.

*Blastocystis* has become an increasingly popular research subject over the last decade, driven in part by its controversial role in gastrointestinal disorders (Poirier et al. 2012). This has resulted in the complete sequencing of the MRO genomes of STs 1, 4 (Pérez-Brocal and Clark 2008), and 7 (Wawrzyniak et al. 2008) and also the nuclear genomes of ST4 (Wawrzyniak et al. 2015) and ST7 (Denoeud et al. 2011), with nuclear genome sequencing data for other STs also available.

The comparative study of MRO genomes from different *Blastocystis* STs is of interest because a number of peculiarities have been noted. These include the lowest repertoire of tRNA genes (*trn*s) seen among mitochondrial genomes of stramenopiles, a longer than expected gene for ribosomal protein S4 (*rps4*) that lacks a start codon, and an unidentified open reading frame that has in-frame stop codons in some STs (Pérez-Brocal and Clark 2008). We here present a comparative analysis incorporating MRO genomes from five additional *Blastocystis* STs in which we explore the distribution, origins, and conservation of these genomic peculiarities.

## Materials and Methods

### Samples, Culture, and DNA Extraction

*Blastocystis* sp. ST2 (strain Flemming from a human host), ST3 [DMP/08-1043, human; DMP/08-326, human; DMP/IH:478, human; ZGR, human (ATCC 50629)], ST6 (SSI:754, human), ST8 (DMP/08-128, *Cercopithecus diana*), and ST9 (F5323, human) were maintained in long term culture in medium LYSGM with 5% adult bovine serum as described (Pérez-Brocal and Clark 2008). ST4 (DMP/10-212, human) was isolated in culture but died out after a short time. Cells in culture were concentrated and partially separated from the bacterial flora using Histopaque 1077 (Sigma-Aldrich Ltd, Gillingham, Dorset, UK) as described (Pérez-Brocal and Clark, 2008).

DNA samples containing the following *Blastocystis* subtypes were also used in this study: ST1 (samples MR14, MR15, MR24, MR25, MR46: *Macaca sylvanus*; SK76, SK95: *Chlorocebus sabaeus*), ST5 (S4-1, pig), ST8 (KE15, human), and ST10 (S3, sheep). DNA was harvested and purified from culture (Pérez-Brocal and Clark 2008), or fecal samples (Alfellani et al. 2013), as previously described. *Blastocystis* ST4 (BT-1) DNA from axenic cultures was purchased from the ATCC (cat# 50608D).

### PCR and Sequencing

Subtype identification was through standard PCR and sequencing of a SSU rDNA region as described (Scicluna et al. 2006). The MRO genome sequences from *Blastocystis* ST2, all ST3s except ZGR and ST4 DMP/10-212 were obtained by “primer walking” and Sanger sequencing as described (Pérez-Brocal and Clark 2008). MRO genome sequences from STs 2, 3 (ZGR), 4 (BT-1), 6, 8, and 9 were obtained from genomic DNA libraries prepared using the Illumina Nextera XT kit, multiplexed on a single flow cell of a HiSeq 2000, with a 2 × 100 bp paired end run.

### Assembly and Annotation

Reads obtained by Sanger sequencing were assembled into a single contig corresponding to the MRO genome using the Staden software package (version 1.7.0, Staden et al. 2000).

For genomes obtained by Illumina sequencing, the CLC Genomics Workbench v.6.5.1 (CLC Bio, Aarhus, Denmark) was used for *de novo* assembly. Raw data were imported as 100bp Illumina paired-end reads with a distance range of 180–250 bp. For the assembly, the standard settings and a contig cut-off size of 2000 bp were used. For identifying MRO genomes in the *de novo* assemblies, a pre-existing MRO genome was “BLASTed” against each assembly. Each MRO contig was then circularized and position 1 was set manually to be the start codon of the *nad3* gene. The resulting sequences were then verified by pairwise alignment to previous MRO genome sequences using the CLC Genomics Workbench proprietary alignment algorithm. The sequences were also aligned using the online ClustalW Multiple Sequence Alignment tool at (http://embnet.vital-it.ch/software/ClustalW.html; last accessed October 2016) (Larkin et al. 2007). Where gaps in the assembly were identified, or where potential anomalies were suspected, the sequence was confirmed using PCR and Sanger sequencing.

The identification of tRNAs and rRNAs and the assignment of protein coding regions and open reading frames were performed by comparison to our previously published and annotated MRO genomes (Pérez-Brocal and Clark 2008). Codon usage bias, pairwise distances, and guanine-cytosine (GC) content were calculated using MEGA (version 6.0) (Tamura et al 2013). The MRO genome sequences determined in this study were deposited into GenBank with the following accession numbers: ST2 Flemming (KU900234/KU900235), ST3 DMP/08-1043 (HQ909887), ST3 DMP/08-326 (HQ909886), ST3 DMP/IH:478 (HQ909888), ST4 DMP/10-212 (KU900236), ST6 SSI:754 (KU900237), ST8 DMP/08-128 (KU900238), and ST9 F5323 (KU90239). The additional *orf160* sequences were deposited with the accession numbers KU900128–KU900130.

### Phylogenetic Analysis of Concatenated *Nad* Genes

Inferred amino acid sequences corresponding to NADH dehydrogenase (nad) subunits from the *Blastocystis* MRO genomes, mitochondrial genomes from other stramenopiles, and from selected other eukaryotes and prokaryotes were extracted from our sequences or downloaded from the NCBI website (http://www.ncbi.nlm.nih.gov). Nine nad subunit protein sequences were concatenated in the order 1, 2, 3, 4, 4L, 5, 6, 7, and 9 (nad1L was excluded because it is not universally found across stramenopile mitochondrial genomes). The final dataset, consisting of 57 taxa, was aligned using MUSCLE, as implemented in MEGA 6.0 (Tamura et al. 2013). Ambiguous regions were trimmed to produce a final alignment consisting of 2854 amino acids. ModelTest, as implemented in MEGA 6.0, was used to select the most appropriate models for phylogenetic analysis. Maximum Likelihood (ML) analysis with 1000 bootstrap replicates was performed using the LG + F + I+ G model with 5 Gamma rate categories in MEGA 6.0. IQ-Tree (Nguyen et al. 2015) was used for ML analysis with a mixture model (LG + C20) of amino acid evolution using with 1000 ultrafast bootstrap replicates (Minh et al. 2013). Bayesian inference was performed using MrBayes (version 3.2; Ronquist et al. 2012) with four Markov chain Monte Carlo (MCMC) strands, 1,000,000 generations, and trees sampled every 100 generations. A consensus tree was produced after excluding an initial burn-in of 25% of the samples, as recommended.

### Analysis of Selection

dN/dS ratios were calculated using the HyPhy package (Pond et al. 2005) implemented at datamonkey.org.

## Results

### Sequencing and Genomic Organization of MROs

The complete MRO genomes of *Blastocystis* ST2, three isolates of ST3 and a divergent isolate of ST4 (DMP/10-212) were sequenced using conventional Sanger sequencing technology, whereas reads corresponding to the MRO genomes of isolates belonging to STs 2, 3, 4, 6, 8, and 9 were extracted from high throughput next generation sequencing (NGS) genome surveys, with finishing using Sanger sequencing where necessary. The genomes of *Blastocystis* ST4 strains BT-1 and WR1 (Wawrzyniak et al. 2015) are almost identical to that of DMP/02-328 whereas ST3 ZGR is identical to the MRO genomes of *Blastocystis* ST3 DMP/08-326 and DMP/IH:478. These three genomes are not discussed separately—when DMP/02-328 is referred to below the same applies to BT-1 and WR1 and when DMP/IH:478 is mentioned, the same information applies to ZGR and DMP/08-326. The ST2 MRO sequence was obtained by both sequencing methods and differed by only a single base call.

The features of the *Blastocytsis* MRO genomes are presented in table 1. All MRO genomes are devoid of introns or repeats and the sizes of the newly sequenced STs do not differ significantly from those sequenced previously. Differences in genome length are accounted for primarily by variations in the intergenic regions (IGRs), which account for between 3.5% and 4.8% of MRO genomes and range in size from 0 to 207 bp with one exception (table 2). A possible location for the mitochondrial DNA origin of replication is between *nad2* and *nad11* in a region of the MRO genome that features a change in the direction of transcription.

**Table 1:** MRO Genome Characteristics of *Blastocystis* sp. Subtypes 1–4 and 6–9

Genome Characteristics	ST 1	ST 2	ST 3	ST 3	ST 3	ST 4	ST 4	ST 6	ST 7	ST8	ST9
	NandII	Flemming	DMP/08-326	DMP/08-1043	DMP/IH:478	DMP/02-328	DMP/10-212	SSI:754	B	DMP/08-128	F5323
GenBank Accession number	EF494740	KU900235	HQ909886	HQ909887	HQ909888	EF494739	KU900236	KU900237	CU914152	KU900238	KU900239
Genome size (bp)	28,385	28,305	28,243	28,268	28,242	27,718	27,817	28,806	29,270	27,958	28,788
Coding density (%)^a^	77.5	78.0	77.5	77.6	77.7	77.1	77.4	77.0	77.1	77.0	77.3
Intergenic spacer (IGS) content (%)	4.1	3.7	3.8	3.9	4.1	3.5	3.6	4.1	4.8	3.7	4.1
Sum of IGS sizes (bp)	1,165	1,044	1,081	1,102	1,160	964	996	1,167	1,399	1,043	1,179
Average size of IGS (bp)	33.3	30.7	30.0	30.6	33.1	36.1	28.5	35.3	37.8	29.8	38.0
Overlapped genes	6	8	7	7	7	8	9	11	7	9	11
G + C content (%)	19.9	19.7	21.6	21.4	21.6	21.9	21.6	18.9	20.1	22.7	18.8

^a^Includes *rps4* and *orf160*.

**Table 2:** Selected Intergenic Region Data from the MRO Genomes of *Blastocystis*

	ST1	ST2	ST3	ST3	ST3	ST4	ST4	ST6	ST7	ST8	ST9
	NandII	Flemming	DMP/08-326	DMP/IH:478	DMP/08-1043	DMP/02-328	DMP/10-212	SSI:754	B	DMP/08-128	F5323
Accession number	EF494740	KU900235	HQ909886	HQ909888	HQ909887	EF494739	KU900236	KU900237	CU914152	KU900238	KU900239
*nad6/rns*	86	85	86	86	88	95	88	94	86	**102**	94
*nad2*/*nad11*^a^	**209**	**216**	**205**	**205**	**207**	**157**	**168**	**191**	**415**	**153**	**190**
*nad5*/*rps11*^a^	54	63	62	62	61	55	59	67	**111**	59	69
*nad4l/trnsF*^b^	**239**	**122**	**104**	**104**	**104**	21	21	19	41	90	15
*trnsN/trnsL*^b^	**151**	**140**	**140**	**140**	**141**	**129**	**133**	**163**	**150**	**131**	**182**
*tRNA^Met^/nad3*	10	9	21	**106**	21	**105**	20	29	22	11	28

^a^Denotes intergenic regions where the direction of transcription changes.

^b^Denotes other regions in which the intergenic distance exceeds 100 bp (bold text) in at least some STs.

**Fig. 1.—:**
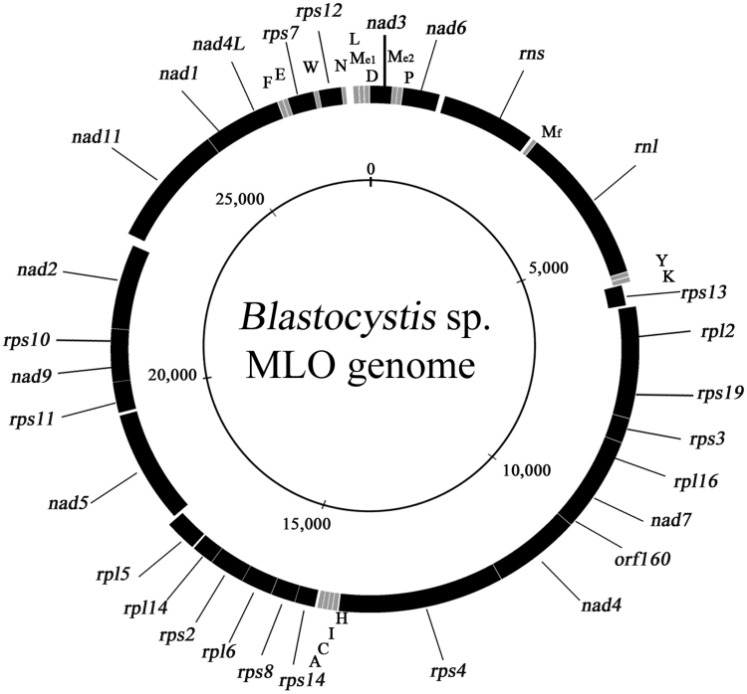
Coding regions of a representative MRO genome of *Blastocystis* sp. Black blocks represent genes that are predicted to be transcribed clockwise (outer ring) or anti-clockwise (inner ring). Grey blocks represent tRNA genes, which are identified by their amino acid using the standard single letter code. Mf and Me1/Me2 are initiator and elongator methionyl tRNAs, respectively. The inner circle shows the approximate size scale. The map is modified from that in Pérez-Brocal and Clark (2008).

The complete repertoire of genes in each genome consists of 27 coding for proteins (one of which, *orf160*, remains unidentified), 2 for rRNAs and 16 for tRNAs. Our gene annotations are in agreement with those published for *Blastocystis* ST7 (Denoeud et al. 2011; Wawrzyniak et al. 2008). A typical *Blastocystis* MRO genome maps as circular with genes on both strands (fig. 1). Gene order is conserved across STs, but the number of overlapping genes can vary even within the same ST (see below).

The total GC content of the MRO genomes is very low (mean 20.75%, range 18.8–22.7%) but GC content is not distributed evenly among genes, with the highest GC content being in tRNA genes (*trns*) and the lowest in ribosomal protein genes (supplementary table S1, Supplementary Material online).

### Phylogenetic Inference

**Fig. 2.—:**
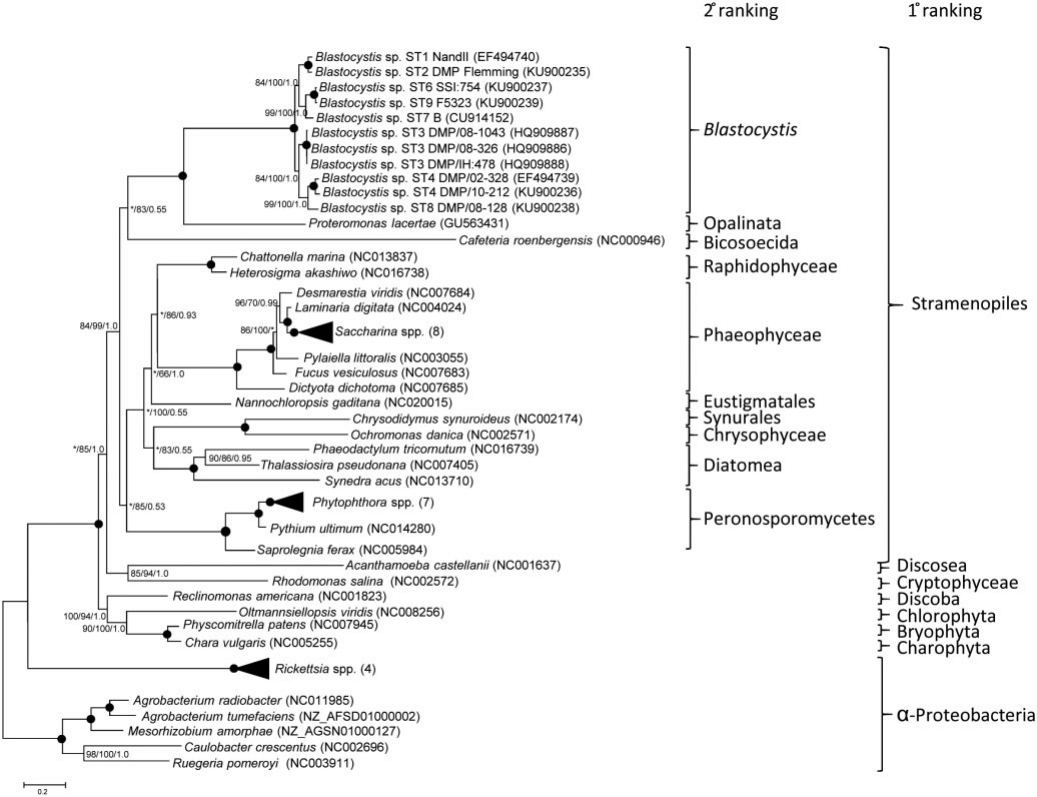
Phylogenetic relationships among the stramenopiles based on concatenated nad genes. Stramenopile species are grouped according to their second highest rank in the taxonomic classification of Adl et al. (2012). The tree shown is that inferred using IQ-Tree analysis as described in the methods section. The Bayesian analysis tree was identical except for the branch order of *Fucus* and *Pylaiella.* Some of the branches have been collapsed to simplify the topology; the number alongside the genus indicates the number of taxa represented. Bootstrap support and posterior probabilities are adjacent to each node in the order Maximum Likelihood/IQ-Tree/Bayesian Analysis. Where boot strap support was 100% and Posterior Probabilities were 1.0, the node is indicated by a filled circle. Bootstrap support lower than 50% and posterior probability values of < 0.5 are indicated by asterisks. GenBank accession numbers of the sequences used are listed in parentheses.

To date, the phylogenetic relationships of *Blastocystis* STs have been determined almost exclusively using SSU rDNA sequences. In order to interpret the origins of MRO genome characteristics it was important to know whether analyses of MRO genome sequences inferred the same relationships. We therefore undertook phylogenetic analyses using concatenated *nad* genes from the *Blastocystis* MRO genomes, and investigated their relationships using the available stramenopile homologues and a selection of other sequences as outgroups. The results are presented in figure 2. Our phylogenetic analysis confirms that within *Blastocystis* there are three major clades, consisting of sequences derived from STs 3/4/8, 6/7/9, and 1/2. The topology is identical to the relationships recovered in trees derived from SSU rDNA analyses (Alfellani et al. 2013).

In SSU rDNA reconstructions, *Blastocystis* is recovered as sister to the Opalinata, a group of non-photosynthetic organisms that includes *Proteromonas, Protoopalina*, and *Karotomorpha* (Cavalier-Smith and Scoble 2013). *Proteromonas lacertae* is the only member of this group for which the relevant sequences are available (Pérez-Brocal et al. 2010). The phylogenetic position of the clade representing *Blastocystis* plus Opalinata is poorly resolved in relation to the remaining stramenopiles. The bicosoecid *Cafeteria* is recovered as representing the most closely related lineage in two of the three analyses but with minimal support. However, this relationship is consistent with recent phylogenomic data analyses (Derelle et al. 2016).

### Codon Usage Bias, tRNAs and Sequence Features of Protein Coding Genes

Codon usage analysis (supplementary table S2, Supplementary Material online) shows that it is highly biased and that between 1 and 8 codons are unused in all but one (ST8) of the *Blastocystis* MRO genomes. Unused and under-used codons are characterized by having G or C in the third and/or second codon position; this is likely linked to the high AT content of the genomes and is typical of mitochondria in general (Knight et al. 2001).

A total of 16 tRNAs are encoded in each *Blastocystis* MRO genome. All are single copy except for an initiator and two non-identical elongator ${\text{tRNA}}_{\text{CAU}}^{\text{Met}}$ genes. No genes encoding tRNA^Gly^, tRNA^Thr^, tRNA^Ser^, tRNA^Gln^, tRNA^Arg^, and tRNA^Val^ are present and are therefore likely to be nuclear encoded and imported from the cytosol (Schneider 2011; Salinas et al. 2012). Leucine is encoded almost exclusively by UUN codons and ${\text{tRNA}}_{\text{TAA}}^{\text{Leu}}$ is the only MRO-encoded leucyl tRNA. A MRO genome-encoded ${\text{tRNA}}_{\text{TAG}}^{\text{Leu}}$ was already missing in the common ancestor of *Blastocystis* and *P*. *lacertae*, and possibly the common ancestor of *Cafeteria* and the Opalinata. Among the other stramenopiles in figure 2, only *Synedra acus* encodes a single ${\text{tRNA}}_{\text{TAA}}^{\text{Leu}}$; the remaining genera are characterized by mitochondrial genomes containing two or even three tRNA^Leu^ genes.

The predicted secondary structures of some tRNA molecules diverge from the standard model structure. These include missing or shortened D-loop and anticodon stems as well as the presence of base mismatches in the stems of several tRNAs (supplementary text, Supplementary Material online). The latter do not necessarily reflect the final structure of the molecules, as the maturation of tRNA *in vivo* involves post-transcriptional modifications, and some of the mismatches we observed do correspond to known modification sites in tRNA (Salinas-Giegé et al. 2015); further, RNA editing cannot be ruled out as modifying the sequences, although there is at present no evidence it is occurring.

ATG is used as an initiation codon in all of the protein coding genes, with the exception of *rps4*, which is discussed later. All three termination codons (TAA, TAG, and TGA) are used, with TAA being by far the most frequent (88.8–100%) (supplementary table S3, Supplementary Material online). TAA termination codons are used exclusively in *P. lacertae* (Pérez-Brocal et al. 2010). As there are in-frame stop codons in *orf160* (discussed later), for clarity, we will use “stop codon” only when referring to the latter and “termination codon” when referring to those at the end of the protein encoding genes.

TGA is used as a termination codon only in ST4, ST6 and ST9 and then to terminate translation of only one gene, *rps19*. Differences in TAG termination codon usage are evident even within subtypes. *rps8* of ST3 is terminated by TAG in one isolate whereas in the others it ends in TAA. In the ST4 genomes, the genes *rps14* and *nad4L* are terminated by TAG in one isolate but TAA in the other (supplementary table S3 and supplementary text, Supplementary Material online).

### Overlapping Genes

All *Blastocystis* MRO genomes contain overlapping genes, a common feature in protist mitochondria (Gray et al. 2004). The number of overlapping genes varies between STs and also between isolates of the same ST (table 3). The number of overlapping nucleotides ranges from 1 to 69 and all overlaps occur between genes encoded on the same strand of DNA. There is a single example of overlap between two *nad* genes (*nad1*–*nad4L*) and this overlap (10 bp) is conserved across STs. In contrast, the overlap between a ribosomal protein gene and a tRNA (*rps12*–*trnsN*) varies from 7 to 69 bp across the genomes. Alignments indicate that a variable number of bases in an oligo-A tract near the 3′ end of *rps12* is responsible for this as it leads to a change of translation frame and therefore length variation of the predicted rps12 protein. In most cases, the degree of variation is much smaller, however. The variation is primarily the result of point mutations creating new start/termination codons near the termini of the coding regions, although in a few cases small insertions are also observed (supplementary text, Supplementary Material online).

**Table 3:** Overlapping Genes and Length of Overlap (in bp) for the Eight MRO Genomes of *Blastocystis*

Isolate	ST1	ST2	ST3	ST3	ST3	ST4	ST4	ST6	ST7	ST8	ST9
	NandII	Flemming	DMP/08-326	DMP/IH:478	DMP/08-1043	DMP/02-328	DMP/10-212	SSI:754	B	DMP/08-128	F5323
GenBank Accession	EF494740	KU900235	HQ909886	HQ909888	HQ909887	EF494739	KU900236	KU900237	CU914152	KU900238	KU900239
*rpl*2–*rps*19	8	8	11	11	11	8	8	8	11	11	8
*rps*19–*rps*3	–	–	–	–	–	4	4	4	–	–	4
*rps3–rpl16*	–	–	–	–	–	–	–	–	–	10	–
*nad*7–*orf*160^a^	56	56	56	55	56	56	56	56	56	56	56
*orf160–nad4*	–	–	–	–	–	–	–	10	–	–	10
*nad*4–*rps*4^b^	–	7	–	–	–	–	11	–	–	–	–
*rps8–rpl6*	–	–	–	–	–	–	–	8	–	–	8
*rpl*6–*rps2*	11	11	11	11	11	11	11	8	11	11	8
*rps2–rpl14*	–	–	–	–	–	–	–	–	–	17	–
*nad*2–*rps*10	–	10	10	10	10	10	10	10	10	10	10
*rps*10–*nad*9	23	23	8	8	8	20	20	23	26	23	20
*nad9–rps11*	–	–	–	–	–	–	–	1	–	–	1
*nad*1–*nad*4L	10	10	10	10	10	10	10	10	10	10	10
*rps*12–tRNA^Asn^	7	38	7	38	38	7	7	38	69	38	69
Total overlap	115	163	113	143	144	126	137	176	193	237	204

^a^The triplet and the single letter amino acid letter designation is given.

^b^The absence in some STs of an initiation codon in *rps4* and the presence of in-frame stop codons in *orf160* complicates the calculation of the overlap lengths involving these genes as it is unclear where the true start of translation is. Accordingly, we have annotated the start of *rps4* as the first triplet after the stop codon of the preceding *nad4* gene and the start of *orf160* as the conserved ATG upstream of the in-frame stop codon(s); the overlap lengths involving these two genes must be viewed as tentative.

### rps4

We identified a putative *rps4* gene in all the MRO genomes. However, *rps4* in ST2, ST4 (DMP/10-212) and ST8 lacks an ATG initiation codon, a trait previously observed in ST1 and ST4 (DMP/02-328) (Pérez-Brocal and Clark 2008). With a length that ranges from 2769 to 3219 bp, the *rps4* gene is double the size of that reported in *P. lacertae* (1473 bp) (Pérez-Brocal et al. 2010); the *P. lacertae rps4* starts with an ATG codon*.* There is also no evidence of a second gene copy when nuclear genomes sequences are searched, so the MRO *rps4* appears to be the only gene for this mitochondrial ribosomal protein.

**Fig. 3.—:**
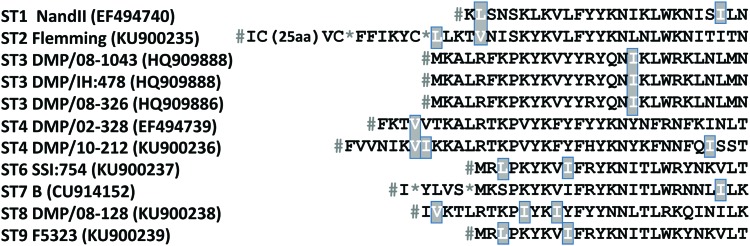
Multiple sequence alignment of the conceptual translation of the N terminus of rps4. Amino acids corresponding to sites of potential alternative initiation codons (TTG/CTG/GTG/ATT) are highlighted as white letters on a grey background. # and * represent the stop codons TAA and TAG respectively. A 25 amino acid sequence in frame has been deleted in ST2 for presentation reasons. No potential alternative initiation codons are found in the deleted residues.

We explored the possibility of alternative start codons in ST1, ST2, ST4, and ST8 (fig. 3). However, it was not possible to identify any alternative start position in the amino acid sequences that aligned across subtypes. In most sequences, the in-frame stop codon upstream of the *rps4* coding region is TAA, the most commonly used termination codon, so that read-through seem an unlikely explanation for the lack of an initiation codon (fig. 3).

### orf160

The only remaining hypothetical protein (designated orf160 for clarity, although the length varies between 158 and 162 amino acids) is encoded in all MRO genomes flanked by *nad4* and *nad7*, overlapping with the latter by 56 nucleotides. It has no sequence similarity to genes or proteins in the public databases even at low thresholds of similarity.

This seemingly genus-specific protein also exhibits the lowest intra-genus amino acid identity (27.5%) of all proteins encoded in the *Blastocystis* MRO genome (supplementary table S4, Supplementary Material online). It has a %GC and predicted amino acid content that is similar to some of the identified ribosomal proteins, suggesting that it may be a divergent ribosomal protein. If expressed, *orf160* would encode a polypeptide that is longer than the predicted size of the smallest identified ribosomal protein, rps7 (143 amino acids).

**Fig. 4.—:**
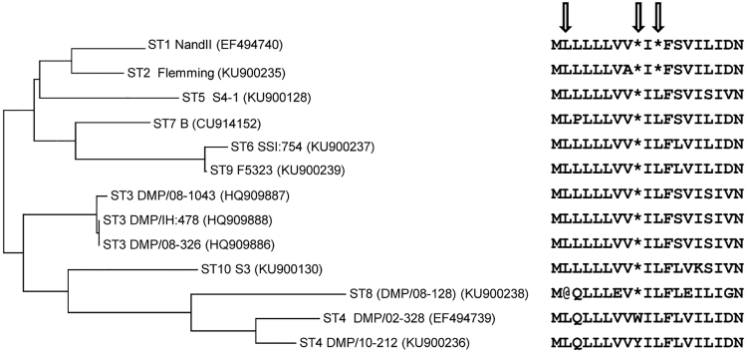
Multiple sequence alignment corresponding to the first 19 amino acids of orf160. The region presented corresponds to the 56 bp overlap of orf160 with nad7. @ = TGA and * = TAG stop codons respectively. The arrows indicate the only positions in which stop codons are seen.

The most unusual feature of this gene is the presence of one or two in-frame stop codons in most of the *orf160* homologues (fig. 4). These include a TAG stop codon at amino acid position 9 in all subtypes except for ST4, where there is TGG (tryptophan, W) in DMP/02-328 and TAT (tyrosine, Y) in DMP/10-212. The sequences in the closely related STs 1 and 2 have an additional TAG stop codon at position 11, which aligns with TTG (Leucine, L) in all other STs. Finally, there is a TGA stop codon at position 2 in ST8 (verified in a second ST8 sample) that aligns with TTA (L) in all other STs.

As for *rps4* (above), the possibility of alternative initiation codons in this region was investigated. Two possible alternative sites were identified, the first a CTG (L) at position 4 and the second a TTG (L) at position 6. However, it is unlikely that these are used for translation initiation in *orf160* as both these sites precede the TAG stop codon at position 9, which is present in all STs except ST4.

## Discussion

All the *Blastocystis* MRO genomes sequenced in this study have an identical gene content and gene order to the three previously reported MRO genomes. Not surprisingly, therefore, most of the genome characteristics reported previously are unchanged: highly biased codon usage, highly reduced tRNA gene complement, etc. Nevertheless, our understanding of three unusual aspects of these genomes has been greatly increased through analysis of the new MRO genome sequences.

The variation in the number of overlapping genes among *Blastocystis* MRO genomes is similar to that seen in the other stramenopile genera in which more than one species has been sequenced. However, we now have greater insight into how the variation in overlap has evolved, being primarily the result of frame shifts and point mutations. We do not yet know what implications these overlaps might have for expression of the encoded proteins. Analysis of *Blastocystis* messenger RNA sequences has to date incorporated a polyA-enrichment step, which may have led to the reduction or exclusion of mitochondrial genome transcripts. We have little information at present on what such transcripts look like and therefore have little insight into how overlapping genes are expressed.

**Fig. 5.—:**
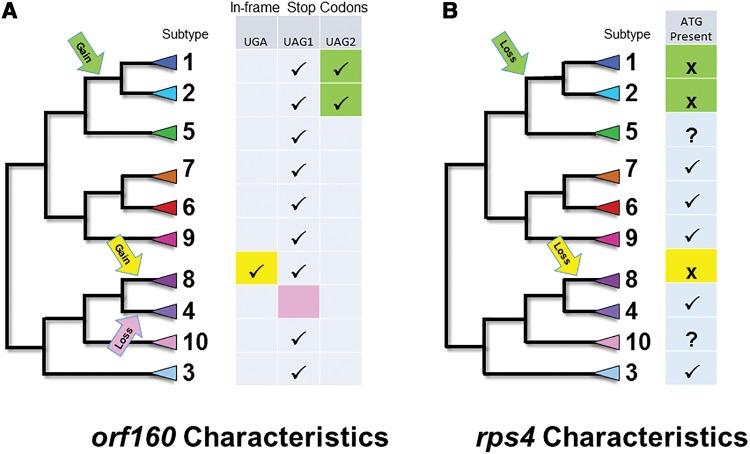
Schematic representation of the proposed gain and loss of stop codons in *orf 160* (*A*) and loss of the start codon in *rps4* (*B*). The cladogram shown is based on Figure 2, with the positions of ST5 and ST10, for which no MRO genome sequence exists, being based on information from SSU rDNA-based trees. Arrows indicate the proposed approximate position of the events under discussion.

The lack of an initiation codon for *rps4* in ST1, ST2, ST4, and ST8 is intriguing, as phylogenetic analyses suggest that the initiation codon either must have been lost independently twice, in the branches leading to ST1/ST2 and to ST4/ST8, or lost earlier in the *Blastocystis* lineage and regained in the branches leading to ST3 and to ST6/ST7/ST9. We believe the former is the most parsimonious interpretation (fig. 5). We are not aware of other examples of convergent loss of an initiation codon.

If the lack of an initiation codon implies loss of function for *rps4*, i.e., that they are pseudogenes, this should only have occurred after transfer of the gene for this essential ribosomal protein to the nuclear genome and acquisition of a targeting signal allowing re-import of the protein product into the MRO. However, we were unable to find a mitochondrial-type *rps4* in the nuclear genomes of ST1 isolate NandII (Gentekaki E et al. unpublished data; Genbank LXWW01000000), ST4 isolate WR1 (Wawrzyniak et al. 2015) or ST7 isolate B (Denoeud et al. 2011), which suggests that such a transfer has not occurred in these STs at least.

To date the only pseudogene in a stramenopile mitochondrial genome has been reported in *Synedra acus*, which has *rps3* and *rps7* genes containing frame shifts and deletions (Ravin et al. 2010); this is very different to what is seen in *rps4* and *orf160* of *Blastocystis*. The *rps4* open reading frame is very long and the only anomaly in the gene is the lack of an initiation codon in some subtypes.

The absence of a nuclear copy implies that the MRO genome *rps4* is functional. In almost all cases the proximal upstream in-frame stop codon is TAA. As this sequence is used for termination of translation of the vast majority of MRO proteins, this suggests that translation of *rps4* must start 3′ to the TAA triplet. However, we were unable to identify a conserved alternative initiation site for translating the protein. There does appear to be a precedent for the occasional use of alternative initiation codons in other stramenopile mitochondrial genomes. The diatom *Phaeodactylum tricornutum* appears to use two different alternative initiation codons (TTG for *cox3*, *cob*, and *tatC* and GTG for *nad7*) as does the alga *Dictyota dichotoma* (TTG for *orf37* and GTG for *rps14*). Elsewhere GTG has been mooted as the initiation codon in the diatom *Synedra acus* for three genes. Finally, ATT and ATA have been proposed as the initiation codon for *atp8* and *nad11* in *Thalassiosira pseudonana*, another diatom. EST data exist for several of these genes, indicating that they are at least transcribed. However, there is no empirical evidence for transcription of *Blastocystis rps4* (nor *orf160*) as there is no coverage of this region of the MRO genome in EST (Stechmann et al. 2008) or RNASeq (Gentekaki E et al. unpublished) data for ST1.

In an attempt to obtain direct evidence for transcription, first strand cDNA from axenically grown *Blastocystis* sp. ST1 (NandII isolate), generated during the study of Stechmann et al. (2008), was used as a template for PCR amplification. Several pairs of primers were designed that targeted either *orf160* or *rps4* using the MRO genome sequence published previously (Pérez-Brocal and Clark 2008), with amplification of *nad7* as a control. Whereas amplification of the control gene was successful, none of the primer pairs designed to amplify *orf160* or *rps4* produced amplification products. However, interpretation of these negative results is difficult, as both genes are extremely A + T-rich (88.6% and 89%, respectively, in NandII), which makes primer design very difficult. The same primer pairs also failed to amplify products using genomic DNA as a template.

Like the long intact open reading frames of *rps4*, the positionally conserved stop codons in the N-terminal coding region of *orf160* do not match the pattern of random mutations expected in a pseudogene, especially given that the gene is not highly conserved overall. The most parsimonious explanation for presence of stop codons in all *orf160* genes but ST4 is that function has been regained (i.e. stop codons have been lost) in the latter lineage (fig. 5). The fact that the in-frame stop codons are TAG and TGA when termination codons are almost exclusively TAA in these AT-rich MRO genomes also suggests that something unusual is occurring.

There is precedent in stramenopiles for TGA-to-tryptophan and the less common TAG-to-leucine termination codon reassignments (Swire et al. 2005; Massey and Garey 2007; Sengupta et al. 2007). TGA-to-tryptophan reassignments have been seen in the bicosoecid *Cafeteria roenbergensis* (GenBank accession NC_000946), and the diatoms *Thalassiosira pseudonana* (Armbrust et al. 2004) and *Skeletonema costatum* (Ehara et al. 2000). Stramenopiles even provide an example of an extremely rare sense-to-nonsense reassignment: the leucine codon TTA has been reassigned as a termination codon in the mitochondrial genome of *Thraustochytrium aureum* (GenBank accession AF288091). However, the reassignment of TAG-to-leucine and/or TGA-to-tryptophan in *Blastocystis* is not straightforward as these triplets are also used as termination codons in the MRO genomes of some STs. A search (Lowe and Eddy 1997) of the ST1 nuclear genome (Gentekaki et al. unpublished data; Genbank LXWW01000000) found no identifiable tRNA genes with anticodons that would allow the decoding of any of the three termination codons. This would be a pre-requisite for codon reassignment to work.

Another potential measure of functionality is whether these genes are under purifying selection. dN/dS ratios were calculated for both *rps4* and *orf160* and compared with those of four other ribosomal protein genes in the MRO genome (*rps3, rps10, rpl6*, and *rpl14*). The dN/dS ratios for *rps4* and *orf160* (0.199 and 0.156, respectively) were comparable to those of the other ribosomal proteins tested (range 0.029–0.210). A ratio of less than 1 is considered an indicator that selection is occurring against non-synonymous substitutions and, although the bias detected is not strong, this evidence also favors functionality of *rps4* and *orf160*.

There are a number of other mechanisms by which both *rps4* and *orf160* could be rendered functional, for example, RNA editing to create initiation codons or remove stop codons, or translational read through to circumvent the stop codons (Beier and Grimm 2001). The available transcript sequences from MRO genomes give no indication that RNA editing is occurring, but as mentioned above the crucial regions of the genomes are absent from the RNA data and attempts to amplify these regions from cDNA were unsuccessful. It is important to know whether the *rps4* and/or *orf160* genes are expressed as mRNAs or proteins, so further investigation of the transcriptome and proteome of this unique organelle is warranted. In addition, the sequencing of MRO genomes from deeper-branching *Blastocystis* STs and species will help to elucidate the origins of the *orf160* and *rps4* anomalies.

## Conclusion

*Blastocystis* MRO genomes show peculiarities related to two genes in particular, *rps4* and *orf160.* These have conventional gene characteristics in some subtypes but others lack an initiation codon (*rps4*) or have stop codons within the reading frame (*orf160*). The absence of any frameshifts and the localization of the peculiarities to the very 5′ end of the genes in both cases are at odds with the pattern of events that would normally accompany pseudogene formation, where random point and indel mutations are the norm. The long uninterrupted reading frame of *rps4* in *Blastocystis* (around 1000 amino acids) on its own suggests it is a functional gene, as does the evidence for purifying selection, and the fact that this is an essential protein of the ribosome and no other copy could be identified in the nuclear genome. Likewise, the use of only rare stop codons (TGA and TAG) in-frame in *orf160*, rather than the common termination codon (TAA), also suggests that this is not a random event. In contrast to the evidence supporting functionality, there is as yet no evidence for transcription or translation of these genes, no evidence of alternative initiation codons, no evidence for RNA editing, and the stop codons found in-frame in *orf160* are also used to terminate translation of other proteins. Further investigation will be required to solve this apparent conundrum.

## Supplementary Material

Supplementary tables S1–S4 and text are available at *Genome Biology and Evolution* online (http://www.gbe.oxfordjournals.org/).

Supplementary Data
