# Supplementary material for: Blastocystis Mitochondrial Genomes Appear to Show Multiple Independent Gains and Losses of Start and Stop Codons
Source: Genome Biol Evol. 2016 Nov 9;8(11):3340–50. doi: 10.1093/gbe/evw255 (PMC5203790; doi:10.1093/gbe/evw255)
Supplement: Supplementary Data [file supp_8_11_3340__index.html]

Blastocystis Mitochondrial Genomes Appear to Show Multiple Independent Gains and Losses of Start and Stop Codons — Supplementary Data 

# *Blastocystis* Mitochondrial Genomes Appear to Show Multiple Independent Gains and Losses of Start and Stop Codons

## Supplementary Data

files

- Supplementary Data - zip file
