# Supplementary material for: Blastocystis Mitochondrial Genomes Appear to Show Multiple Independent Gains and Losses of Start and Stop Codons
Source: Genome Biol Evol. 2016 Nov 9;8(11):3340–50. doi: 10.1093/gbe/evw255 (PMC5203790; doi:10.1093/gbe/evw255)
Supplement: Supplementary Data [file supp_evw255_suppl_data.zip › Supplementary_text_S1.docx]

Supplementary text – Additional information relating to sections in the main text.

**tRNA genes**

Predicted secondary structures of the tRNA molecules that diverge from the standard model were noted. These include initiator tRNA^Met^_CAU_ in which base pairs in the D-loop stem ([Rich and RajBhandary 1976](#_ENREF_32)) are absent, resulting in a stem comprising 2 rather than the conventional 3-4 base pairs. The D loop stem is also short (3 base pairs) in the equivalent tRNA of *Proteromonas lacertae*. A shorter than expected anticodon stem (comprising 4 base pairs) in tRNA^Glu^ was noted in all MLO genomes except those of ST4 and ST8, in which the anticodon stem consists of the usual 5 base pairs. A number of non-Watson-Crick base pairings were also predicted in the tRNAs. These include a C-A mismatch in the acceptor stem of tRNA^Lys^ that is only present in ST1 and ST2, and U-U mismatches in the acceptor stem of both tRNA^Pro^ and tRNA^Trp^, in the T-stem loop of tRNA^Cys^ and in the anticodon stem-loop of elongator tRNA^Met1^ of all subtypes. These unusual predicted secondary structures do not necessarily reflect the final structure of the molecules, as post transcriptional removal of mismatches has been reported in other organisms ([Laforest et al. 1997](#_ENREF_18); [Forget et al. 2002](#_ENREF_11); [Fu et al. 2014](#_ENREF_12)).

**Termination codons**

TGA is used as a termination codon only in *Blastocystis* only to terminate translation of *rps19* in three subtypes. There are a number of other stramenopile mitogenomes in which TGA is not used at all, including those of *Saprolegnia ferax*, *Thraustotheca clavata*, *Achlya hypogyna*, *Pythium ultimum*, *Chrysodidymus synuroides*, *Ochromonas danica* and *Phaeodactylum tricornutum.* Note that this observation is based on only a single genome from each genus, however. In the genus *Nannochloropsis,* where mitogenomes from six species have been sequenced, only one (*N. oculata*) uses TGA as a termination codon and then only once (see supplementary table S4). Differences in TAG termination codon usage are evident at isolate level within *Blastocystis* ST3 and ST4. *rps8* of ST3, which is the same length in all three genomes (402 bp), is terminated by TAG in one while in the others it ends in TAA. In the ST4 genomes, the genes *rps14* and *nad4L* are terminated by TAG in DMP/10-212 but TAA in DMP/02-328.

**Overlapping genes**

Gene overlap is a common feature in protist mitochondria ([Gray et al. 2004](#_ENREF_13)) and can be divided into two categories. The first consists of overlapping genes that occur on the same strand and orientation and often, but not always, in a different reading frame. These are considered “true” overlaps as the same RNA transcript is generated ([Labuschagne et al. 2014](#_ENREF_17)). The second type of overlap occurs between genes on different strands of the mitogenome and involve the generation of different RNA transcripts ([Labuschagne et al. 2014](#_ENREF_17)). We noted that all the stramenopile mitogenomes used for the *nad* gene phylogenetic analysis in **Figure 2** have at least one pair of overlapping genes.

The number of overlapping genes varies between *Blastocystis* STs and also between isolates of the same ST (**Table 3**). For comparison, we examined overlapping genes in the mitogenomes of those stramenopile genera in which more than one species has been sequenced (*Phytotophora, Saccharina, Nannochloropsis* and *Sargassum* spp*.*) (supplementary Table S3). The number of overlapping genes ranges from 2-14 and there is some variation in the overlap in certain species within genera. Overlap lengths range from 1-71bp and, as in *Blastocystis*, generally occur between genes encoded on the same strand. The majority of overlaps occur between ribosomal protein genes or between ribosomal protein and *nad* genes.

Where possible, we attempted to determine through multiple sequence alignments the origin of the differences between *Blastocystis* STs. We found that the variable length of overlap (8 or 11bp) between *rpl2*-*rps19* arises from the insertion of three bases resulting in an additional amino acid in the C terminus of rpl2 in all ST3 isolates, ST7 and ST8. The 4 bp overlap between *rps19* and *rps3* corresponds to the sequence ATGA in which the first 3 nucleotides represents the initiation codon of *rps3* and the latter three the termination codon of *rps19.* This is unique to the ST4 isolates, ST6 and ST9 because only these STs use TGA stop codons. In all other STs the *rps3* start codon immediately follows the termination codon of *rps19*. A similar situation arises in the overlap between *rps8* and *rpl6,* which is specific to ST6 and ST9. Here the overlap sequence is ATGTATAA; the initiation and termination triplets of the two genes are separated by two bp.

Elsewhere in the MLOs, point mutations in the 3’ end of *rps10* that generate stop codons are responsible for the observed differences in overlap between *rps10* and *nad9* across STs. Conversely, absence of the *nad2*-*rps10* overlap in ST1 is due to a new start codon being used for *rps10*, the original start codon now being out of frame with the rest of *rps10;* the sequence around the ‘original’ start codon is still conserved with the other STs as it is part of the nad2 coding region. The overall effect of this mutation is the N-terminal truncation of rps10 in ST1; we verified that this feature is present in other ST1 isolates (GenBank accessions KU900131-KU900137).

Some overlaps unique to ST8 were found, namely the overlaps *rps2*-*rpl14* (17bp) and *rps3*-*rpl6* (10bp). In the former case the *rpl14* coding region is the same length, 396 bp, as in ST4 (the most closely related *Blastocystis* subtype), but *rps2* contains a 21bp insertion near the 3’ end of the gene resulting in an overlap that is not present in other subtypes and a coding region terminating in TAG rather than TAA. The overlap of 10 bp between *rps3* and *rpl6* was also found to occur because the *rps3* gene in ST8 has a short insertion near the 3’ end of the gene when compared to all other STs.

References

Forget L, Ustinova J, Wang Z, Huss VAR, Lang BF. 2002. *Hyaloraphidium curvatum*: a linear mitochondrial genome, tRNA editing, and an evolutionary link to lower fungi. Mol Biol Evol. 19: 310-319.

Fu C-J, Sheikh S, Miao W, Andersson SGE, Baldauf SL. 2014. Missing genes, multiple ORFs, and C-to-U type RNA Editing in *Acrasis kona* (Heterolobosea, Excavata) mitochondrial DNA. Genome Biol Evol. 6: 2240-2257.

Gray MW, Lang BF, Burger G. 2004. Mitochondria of protists. Ann Rev Genet. 381: 477-524.

Labuschagne C, Kotzé A, Grobler JP, Dalton DL. 2014. The complete sequence of the mitochondrial genome of the African Penguin (*Spheniscus demersus*) Gene 534: 113-118.

Laforest, M-J, Roewer I, Lang BF. 1997. Mitochondrial tRNAs in the lower fungus *Spizellomyces* *punctatus.* tRNA editing and UAG ‘Stop’ codons recognized as leucine. Nucleic Acids Res. 25: 626-632.

Rich A, RajBhandary UL. 1976. Transfer RNA: molecular structure, sequence, and properties. Ann Rev Biochem. 45: 805-860.
