# Supplementary material for: Blastocystis Mitochondrial Genomes Appear to Show Multiple Independent Gains and Losses of Start and Stop Codons
Source: Genome Biol Evol. 2016 Nov 9;8(11):3340–50. doi: 10.1093/gbe/evw255 (PMC5203790; doi:10.1093/gbe/evw255)
Supplement: Supplementary Data [file supp_evw255_suppl_data.zip › Supplementary_Table_S1.pdf]

	ST1 NandII	ST2 Flemming	ST3 DMP/08-326	ST3 DMP/08-1043	ST3 IH:478	ST4 DMP/02-328	ST4 DMP/10-212	ST6 SSI:754	ST7 B	ST8 DMP/08-128	ST9 F5323
Locus	G + C	G + C	G + C	G + C	G + C	G + C	G + C	G + C	G + C	G + C	G + C
nad3	25.6	24.2	26.1	25.8	26.1	25.0	22.8	25.0	25.0	24.7	24.2
Met tRNA	27.8	27.8	32.0	32.0	32.0	28.8	32.0	31.9	29.2	30.6	30.6
Pro tRNA	39.8	38.3	38.9	38.9	38.9	38.3	38.9	34.2	34.3	38.4	34.2
nad6	17.3	16.8	19.4	19.4	19.5	20.3	20.1	17.2	17.7	19.1	17.3
rns	29.3	27.5	27.6	28.0	27.8	27.7	26.5	27.1	26.5	28.3	27.6
Met tRNA	31.3	29.8	29.8	29.8	29.8	28.3	29.8	29.9	29.8	31.3	29.9
rnl	28.7	28.3	28.8	25.4	28.7	27.6	28.0	27.9	28.5	27.7	27.9
Tyr tRNA	39.5	39.5	43.0	43.0	43.0	39.1	43.0	39.1	39.1	41.2	39.1
Lys tRNA	43.2	43.2	40.3	40.3	40.3	36.5	40.3	41.9	37.8	35.1	40.5
rps13	14.9	14.3	18.0	16.2	18.0	16.8	19.0	11.5	14.7	18.7	13.5
rpl2	23.2	23.5	25.8	26.2	25.5	27.4	26.2	23.3	23.8	28.6	22.9
rps19	18.4	14.9	17.3	18.1	17.6	19.8	16.0	16.9	18.9	17.3	16.1
rps3	10.9	12.3	14.3	15.2	14.6	17.1	16.2	11.2	12.8	17.5	10.9
rpl16	20.7	20.7	22.9	23.1	22.9	25.7	24.3	20.4	21.2	26.0	20.4
nad7	26.2	26.7	27.3	27.6	27.4	27.8	27.7	26.2	27.1	28.7	26.5
orf160	11.4	13.0	14.5	15.6	15.3	16.9	17.8	11.7	13.3	19.3	11.0
nad4	21.1	21.4	21.3	21.1	21.4	21.0	20.6	20.7	22.5	21.9	20.8
rps4	11.0	10.8	14.9	14.7	14.9	15.0	15.3	10.5	11.6	18.3	9.5
His tRNA	31.1	29.7	29.2	27.8	29.2	28.4	29.2	27.4	28.4	29.7	27.4
Ile tRNA	36.5	37.9	40.3	40.3	40.3	33.8	40.3	32.4	33.8	32.4	32.4
Cys tRNA	27.4	27.4	27.8	27.8	27.8	27.4	27.8	27.4	27.4	27.4	27.4
Ala tRNA	26.4	30.6	27.8	27.8	27.8	27.8	27.8	27.8	29.2	27.8	27.8
rps14	14.2	13.9	18.5	20.0	18.5	18.8	17.0	10.5	15.2	18.9	12.4
rps8	16.4	14.6	17.2	18.2	17.4	21.1	22.1	14.8	15.4	22.8	15.1
rpl6	10.9	12.1	15.7	14.0	15.7	14.9	15.6	10.8	12.8	16.0	10.6
rps2	11.4	10.9	12.5	13.2	12.7	16.6	16.9	11.3	13.2	18.9	10.8
rpl14	17.2	15.9	18.6	18.7	18.2	19.7	18.2	16.5	17.9	18.7	16.5
rpl5	11.6	10.6	14.5	13.4	14.3	16.1	16.5	9.4	11.6	15.0	9.0
nad5	25.1	23.9	25.3	25.3	25.3	25.1	23.7	22.4	24.4	25.8	23.0
rps11	14.6	14.4	17.1	16.6	17.2	19.7	20.0	14.0	15.2	20.8	14.5
nad9	20.3	19.5	20.8	20.3	20.8	22.7	22.0	20.5	20.6	24.7	20.0
rps10	11.1	10.8	18.2	18.8	17.6	19.2	18.8	11.6	13.3	18.2	12.1
nad2	16.7	16.7	18.2	17.9	18.0	17.7	17.1	15.9	18.0	18.6	15.6
nad11	20.3	19.8	22.8	22.4	22.8	22.0	22.7	19.0	21.8	22.4	19.0
nad1	27.3	26.9	27.5	27.1	27.5	27.4	27.4	26.2	26.0	28.0	26.0
nad4L	17.8	16.3	16.1	15.5	15.9	16.2	15.1	16.2	16.7	18.2	16.5
Phe tRNA	37	37.0	41.7	41.7	41.7	37.0	41.7	37.0	37.0	39.7	38.3
Glu tRNA	40.3	40.3	40.3	40.3	40.3	38.9	40.3	41.7	40.3	40.3	40.3
rps7	12.6	13.2	14.3	13.4	14.3	15.0	15.7	13.2	11.6	15.5	13.2
Trp tRNA	29.6	29.6	32.4	32.4	32.4	28.2	32.4	31.0	33.8	29.6	31.0
rps12	25.4	27.2	26.2	26.2	27.2	28.8	26.7	25.7	26.3	29.4	26.3
Asn tRNA	30.5	30.5	33.3	33.3	33.3	34.7	33.3	35.6	35.6	34.2	35.3
Leu tRNA	31.4	31.4	33.3	33.3	33.3	28.6	33.3	30.0	32.2	30.5	30.5
Met tRNA	33.8	33.8	34.7	34.7	34.7	33.8	34.7	33.8	33.8	33.8	32.4
Asp tRNA	36.5	36.5	31.9	31.9	31.9	29.7	31.9	33.8	39.0	29.3	33.8

Supplementary Table S1. G+C content of individual genes across *Blastocystis* mitochondrial genomes.
