# Supplementary material for: Blastocystis Mitochondrial Genomes Appear to Show Multiple Independent Gains and Losses of Start and Stop Codons
Source: Genome Biol Evol. 2016 Nov 9;8(11):3340–50. doi: 10.1093/gbe/evw255 (PMC5203790; doi:10.1093/gbe/evw255)
Supplement: Supplementary Data [file supp_evw255_suppl_data.zip › Supplementary_Table_S2.docx]

Supplementary Table S2: Codon Usage in *Blastocystis* sp. Subtypes

1. Summary of unused codons in individual *Blastocystis* MRO genomes.

| *Blastocystis* sp. | GenBank Accession number | Unused codon^a^ |
| --- | --- | --- |
| ST1 NandII | EF494740 | UGA(Term); ACG(T); AGG(R); CAG(Q); CCC(P); CGG(R); GAG(E) and GGG(G) |
| ST2 Flemming | KU900235 | UGA(Term); CAG(Q); CCC(P); CGG(R) and GGG(G) |
| ST3 DMP/08-326 | HQ909886 | UGA(Term); ACG(T); AGG(R); CUG(L); CCC(P); CGG(R) and GGG(G) |
| ST3 DMP/08-1043 | HQ909887 | UGA(Term); CGG(R); AGG(R); GGG(G) |
| ST3 DMP/IH:478 | HQ909888 | UGA(Term); AGG(R); CCC(P); CGG(R); GAG(E) and GGG(G) |
| ST4 DMP/02-328 | EF494739 | GAG(E) |
| ST4 DMP/10-212 | KU900236 | UAG(Term) |
| ST6 SSI:754 | KU900237 | CAG(Q); CUC(L); CCC(P); CGG(R) and GGG(G) |
| ST7 B | CU914152 | UGA(Term); ACG(T); AGG(R); CAG(Q); CCG(P); CGC(R); CGG(R); and GAG(E) |
| ST8 DMP/08-128 | KU900238 | All codons used |
| ST9 F5323 | KU900239 | AGG(R); CCG(P) and CGG(R) |

B. Codon usage in individual *Blastocystis* MRO sequences. The relative synonymous codon usage (RSCU) for the coding sequences in *Blastocystis* sp. STs 1-4, and 6-9 were calculated using MEGA (version 6.0; Tamura et al 2013). Termination codons and/or stop codons (which are seen in *orf160* in some STs) are indicated by asterisks.

|  | **Codon** | **Count** | **RSCU** | **Codon** | **Count** | **RSCU** | **Codon** | **Count** | **RSCU** | **Codon** | **Count** | **RSCU** |
| --- | --- | --- | --- | --- | --- | --- | --- | --- | --- | --- | --- | --- |
| **U** | UUU(F) | 487 | 1.73 | UCU(S) | 211 | 2.94 | UAU(Y) | 570 | 1.91 | UGU(C) | 82 | 1.74 |
|  | UUC(F) | 77 | 0.27 | UCC(S) | 9 | 0.13 | UAC(Y) | 26 | 0.09 | UGC(C) | 12 | 0.26 |
|  | UUA(L) | 810 | 5.38 | UCA(S) | 73 | 1.02 | UAA(*) | 26 | 2.69 | UGA(*) | 0 | 0 |
|  | UUG(L) | 22 | 0.15 | UCG(S) | 1 | 0.01 | UAG(*)^†^ | 3 | 0.31 | UGG(W) | 42 | 1 |
| **C** | CUU(L) | 63 | 0.42 | CCU(P) | 60 | 1.7 | CAU(H) | 78 | 1.86 | CGU(R) | 87 | 2.58 |
|  | CUC(L) | 4 | 0.03 | CCC(P) | 0 | 0 | CAC(H) | 6 | 0.14 | CGC(R) | 1 | 0.03 |
|  | CUA(L) | 3 | 0.02 | CCA(P) | 80 | 2.27 | CAA(Q) | 163 | 2 | CGA(R) | 11 | 0.33 |
|  | CUG(L) | 1 | 0.01 | CCG(P) | 1 | 0.03 | CAG(Q) | 0 | 0 | CGG(R) | 0 | 0 |
| **A** | AUU(I) | 559 | 1.89 | ACU(T) | 189 | 2.21 | AAU(N) | 779 | 1.89 | AGU(S) | 131 | 1.82 |
|  | AUC(I) | 39 | 0.13 | ACC(T) | 14 | 0.16 | AAC(N) | 46 | 0.11 | AGC(S) | 6 | 0.08 |
|  | AUA(I) | 288 | 0.98 | ACA(T) | 139 | 1.63 | AAA(K) | 717 | 1.99 | AGA(R) | 103 | 3.06 |
|  | AUG(M) | 131 | 1 | ACG(T) | 0 | 0 | AAG(K) | 5 | 0.01 | AGG(R) | 0 | 0 |
| **G** | GUU(V) | 175 | 2.15 | GCU(A) | 131 | 2.34 | GAU(D) | 195 | 1.99 | GGU(G) | 256 | 3.91 |
|  | GUC(V) | 13 | 0.16 | GCC(A) | 3 | 0.05 | GAC(D) | 1 | 0.01 | GGC(G) | 4 | 0.06 |
|  | GUA(V) | 135 | 1.66 | GCA(A) | 88 | 1.57 | GAA(E) | 170 | 2 | GGA(G) | 2 | 0.03 |
|  | GUG(V) | 3 | 0.04 | GCG(A) | 2 | 0.04 | GAG(E) | 0 | 0 | GGG(G) | 0 | 0 |
|  | **U** | | | **C** | | | **A** | | | **G** | | |

**Table S2Ba**. Codon usage in *Blastocystis* sp. ST1 NandII (EF494740).

^†^Includes two in-frame stop codons in *orf160*.

|  | **Codon** | **Count** | **RSCU** | **Codon** | **Count** | **RSCU** | **Codon** | **Count** | **RSCU** | **Codon** | **Count** | **RSCU** |
| --- | --- | --- | --- | --- | --- | --- | --- | --- | --- | --- | --- | --- |
| **U** | UUU(F) | 517 | 1.8 | UCU(S) | 225 | 3.06 | UAU(Y) | 573 | 1.92 | UGU(C) | 90 | 1.86 |
|  | UUC(F) | 57 | 0.2 | UCC(S) | 8 | 0.11 | UAC(Y) | 23 | 0.08 | UGC(C) | 7 | 0.14 |
|  | UUA(L) | 802 | 5.25 | UCA(S) | 72 | 0.98 | UAA(*) | 27 | 1.86 | UGA(*) | 0 | 0 |
|  | UUG(L) | 36 | 0.24 | UCG(S) | 1 | 0.01 | UAG(*)^†^ | 2 | 0.14 | UGG(W) | 42 | 2 |
| **C** | CUU(L) | 68 | 0.45 | CCU(P) | 74 | 2.13 | CAU(H) | 82 | 1.91 | CGU(R) | 79 | 2.44 |
|  | CUC(L) | 1 | 0.01 | CCC(P) | 0 | 0 | CAC(H) | 4 | 0.09 | CGC(R) | 8 | 0.25 |
|  | CUA(L) | 8 | 0.05 | CCA(P) | 63 | 1.81 | CAA(Q) | 151 | 2 | CGA(R) | 8 | 0.25 |
|  | CUG(L) | 1 | 0.01 | CCG(P) | 2 | 0.06 | CAG(Q) | 0 | 0 | CGG(R) | 0 | 0 |
| **A** | AUU(I) | 529 | 1.77 | ACU(T) | 183 | 2.23 | AAU(N) | 802 | 1.9 | AGU(S) | 127 | 1.73 |
|  | AUC(I) | 28 | 0.09 | ACC(T) | 9 | 0.11 | AAC(N) | 41 | 0.1 | AGC(S) | 8 | 0.11 |
|  | AUA(I) | 339 | 1.14 | ACA(T) | 135 | 1.65 | AAA(K) | 711 | 1.96 | AGA(R) | 98 | 3.03 |
|  | AUG(M) | 127 | 1 | ACG(T) | 1 | 0.01 | AAG(K) | 14 | 0.04 | AGG(R) | 1 | 0.03 |
| **G** | GUU(V) | 173 | 2.11 | GCU(A) | 143 | 2.49 | GAU(D) | 186 | 1.95 | GGU(G) | 248 | 3.86 |
|  | GUC(V) | 16 | 0.2 | GCC(A) | 3 | 0.05 | GAC(D) | 5 | 0.05 | GGC(G) | 6 | 0.09 |
|  | GUA(V) | 132 | 1.61 | GCA(A) | 83 | 1.44 | GAA(E) | 169 | 1.99 | GGA(G) | 3 | 0.05 |
|  | GUG(V) | 7 | 0.09 | GCG(A) | 1 | 0.02 | GAG(E) | 1 | 0.01 | GGG(G) | 0 | 0 |
|  | **U** | | | **C** | | | **A** | | | **G** | | |

**Table S2Bb**. Codon usage in *Blastocystis* sp. ST2 Flemming (KU900235)

^†^Includes two in-frame stop codons in *orf160.*

|  | **Codon** | **Count** | **RSCU** | **Codon** | **Count** | **RSCU** | **Codon** | **Count** | **RSCU** | **Codon** | **Count** | **RSCU** |
| --- | --- | --- | --- | --- | --- | --- | --- | --- | --- | --- | --- | --- |
| **U** | UUU(F) | 542 | 1.75 | UCU(S) | 192 | 2.58 | UAU(Y) | 499 | 1.9 | UGU(C) | 88 | 1.69 |
|  | UUC(F) | 78 | 0.25 | UCC(S) | 4 | 0.05 | UAC(Y) | 27 | 0.1 | UGC(C) | 16 | 0.31 |
|  | UUA(L) | 772 | 5.11 | UCA(S) | 100 | 1.34 | UAA(*) | 25 | 2.68 | UGA(*) | 0 | 0 |
|  | UUG(L) | 36 | 0.24 | UCG(S) | 3 | 0.04 | UAG(*)^†^ | 3 | 0.32 | UGG(W) | 42 | 1 |
| **C** | CUU(L) | 90 | 0.6 | CCU(P) | 68 | 1.84 | CAU(H) | 92 | 1.82 | CGU(R) | 146 | 4.04 |
|  | CUC(L) | 1 | 0.01 | CCC(P) | 4 | 0.11 | CAC(H) | 9 | 0.18 | CGC(R) | 10 | 0.28 |
|  | CUA(L) | 7 | 0.05 | CCA(P) | 72 | 1.95 | CAA(Q) | 195 | 1.98 | CGA(R) | 9 | 0.25 |
|  | CUG(L) | 1 | 0.01 | CCG(P) | 4 | 0.11 | CAG(Q) | 2 | 0.02 | CGG(R) | 0 | 0 |
| **A** | AUU(I) | 614 | 2.27 | ACU(T) | 287 | 2.61 | AAU(N) | 701 | 1.92 | AGU(S) | 141 | 1.89 |
|  | AUC(I) | 34 | 0.13 | ACC(T) | 13 | 0.12 | AAC(N) | 30 | 0.08 | AGC(S) | 7 | 0.09 |
|  | AUA(I) | 164 | 0.61 | ACA(T) | 139 | 1.26 | AAA(K) | 621 | 1.98 | AGA(R) | 52 | 1.44 |
|  | AUG(M) | 150 | 1 | ACG(T) | 1 | 0.01 | AAG(K) | 6 | 0.02 | AGG(R) | 0 | 0 |
| **G** | GUU(V) | 238 | 2.84 | GCU(A) | 139 | 2.22 | GAU(D) | 196 | 1.98 | GGU(G) | 243 | 3.72 |
|  | GUC(V) | 12 | 0.14 | GCC(A) | 6 | 0.1 | GAC(D) | 2 | 0.02 | GGC(G) | 10 | 0.15 |
|  | GUA(V) | 81 | 0.97 | GCA(A) | 101 | 1.61 | GAA(E) | 168 | 1.99 | GGA(G) | 8 | 0.12 |
|  | GUG(V) | 4 | 0.05 | GCG(A) | 5 | 0.08 | GAG(E) | 1 | 0.01 | GGG(G) | 0 | 0 |
|  | **U** | | | **C** | | | **A** | | | **G** | | |

**Table S2Bc**. Codon usage in *Blastocystis* sp. ST3 DMP/08-1043 (HQ909887)

^†^Includes in-frame stop codon in *orf160*

|  | **Codon** | **Count** | **RSCU** | **Codon** | **Count** | **RSCU** | **Codon** | **Count** | **RSCU** | **Codon** | **Count** | **RSCU** |
| --- | --- | --- | --- | --- | --- | --- | --- | --- | --- | --- | --- | --- |
| **U** | UUU(F) | 537 | 1.75 | UCU(S) | 198 | 2.66 | UAU(Y) | 499 | 1.88 | UGU(C) | 93 | 1.81 |
|  | UUC(F) | 78 | 0.25 | UCC(S) | 9 | 0.12 | UAC(Y) | 32 | 0.12 | UGC(C) | 10 | 0.19 |
|  | UUA(L) | 787 | 5.18 | UCA(S) | 86 | 1.15 | UAA(*) | 26 | 2.79 | UGA(*) | 0 | 0 |
|  | UUG(L) | 27 | 0.18 | UCG(S) | 3 | 0.04 | UAG(*)^†^ | 2 | 0.21 | UGG(W) | 43 | 1 |
| **C** | CUU(L) | 90 | 0.59 | CCU(P) | 66 | 1.77 | CAU(H) | 99 | 1.83 | CGU(R) | 138 | 3.8 |
|  | CUC(L) | 3 | 0.02 | CCC(P) | 0 | 0 | CAC(H) | 9 | 0.17 | CGC(R) | 13 | 0.36 |
|  | CUA(L) | 4 | 0.03 | CCA(P) | 79 | 2.12 | CAA(Q) | 191 | 1.97 | CGA(R) | 11 | 0.3 |
|  | CUG(L) | 1 | 0.01 | CCG(P) | 4 | 0.11 | CAG(Q) | 3 | 0.03 | CGG(R) | 0 | 0 |
| **A** | AUU(I) | 604 | 2.25 | ACU(T) | 277 | 2.55 | AAU(N) | 689 | 1.89 | AGU(S) | 141 | 1.89 |
|  | AUC(I) | 40 | 0.15 | ACC(T) | 17 | 0.16 | AAC(N) | 40 | 0.11 | AGC(S) | 10 | 0.13 |
|  | AUA(I) | 160 | 0.6 | ACA(T) | 141 | 1.3 | AAA(K) | 613 | 1.98 | AGA(R) | 56 | 1.54 |
|  | AUG(M) | 151 | 1 | ACG(T) | 0 | 0 | AAG(K) | 6 | 0.02 | AGG(R) | 0 | 0 |
| **G** | GUU(V) | 231 | 2.7 | GCU(A) | 133 | 2.15 | GAU(D) | 195 | 1.97 | GGU(G) | 239 | 3.71 |
|  | GUC(V) | 19 | 0.22 | GCC(A) | 5 | 0.08 | GAC(D) | 3 | 0.03 | GGC(G) | 11 | 0.17 |
|  | GUA(V) | 88 | 1.03 | GCA(A) | 105 | 1.7 | GAA(E) | 168 | 1.99 | GGA(G) | 8 | 0.12 |
|  | GUG(V) | 4 | 0.05 | GCG(A) | 4 | 0.06 | GAG(E) | 1 | 0.01 | GGG(G) | 0 | 0 |
|  | **U** | | | **C** | | | **A** | | | **G** | | |

**Table S2Bd**. Codon usage in *Blastocystis* sp. ST3 DMP/08-326 (HQ909886)

^†^Includes in-frame stop codon in *orf160*.

|  | **Codon** | **Count** | **RSCU** | **Codon** | **Count** | **RSCU** | **Codon** | **Count** | **RSCU** | **Codon** | **Count** | **RSCU** |
| --- | --- | --- | --- | --- | --- | --- | --- | --- | --- | --- | --- | --- |
| **U** | UUU(F) | 540 | 1.75 | UCU(S) | 198 | 2.65 | UAU(Y) | 498 | 1.88 | UGU(C) | 94 | 1.81 |
|  | UUC(F) | 77 | 0.25 | UCC(S) | 9 | 0.12 | UAC(Y) | 33 | 0.12 | UGC(C) | 10 | 0.19 |
|  | UUA(L) | 785 | 5.19 | UCA(S) | 87 | 1.16 | UAA(*) | 26 | 2.79 | UGA(*) | 0 | 0 |
|  | UUG(L) | 28 | 0.19 | UCG(S) | 3 | 0.04 | UAG(*)^†^ | 2 | 0.21 | UGG(W) | 42 | 1 |
| **C** | CUU(L) | 88 | 0.58 | CCU(P) | 65 | 1.74 | CAU(H) | 99 | 1.83 | CGU(R) | 138 | 3.76 |
|  | CUC(L) | 2 | 0.01 | CCC(P) | 0 | 0 | CAC(H) | 9 | 0.17 | CGC(R) | 14 | 0.38 |
|  | CUA(L) | 4 | 0.03 | CCA(P) | 79 | 2.12 | CAA(Q) | 191 | 1.97 | CGA(R) | 11 | 0.3 |
|  | CUG(L) | 1 | 0.01 | CCG(P) | 5 | 0.13 | CAG(Q) | 3 | 0.03 | CGG(R) | 0 | 0 |
| **A** | AUU(I) | 605 | 2.25 | ACU(T) | 274 | 2.53 | AAU(N) | 692 | 1.89 | AGU(S) | 142 | 1.9 |
|  | AUC(I) | 42 | 0.16 | ACC(T) | 18 | 0.17 | AAC(N) | 39 | 0.11 | AGC(S) | 10 | 0.13 |
|  | AUA(I) | 161 | 0.6 | ACA(T) | 141 | 1.3 | AAA(K) | 612 | 1.98 | AGA(R) | 57 | 1.55 |
|  | AUG(M) | 151 | 1 | ACG(T) | 1 | 0.01 | AAG(K) | 7 | 0.02 | AGG(R) | 0 | 0 |
| **G** | GUU(V) | 233 | 2.71 | GCU(A) | 133 | 2.15 | GAU(D) | 195 | 1.96 | GGU(G) | 239 | 3.71 |
|  | GUC(V) | 19 | 0.22 | GCC(A) | 5 | 0.08 | GAC(D) | 4 | 0.04 | GGC(G) | 11 | 0.17 |
|  | GUA(V) | 89 | 1.03 | GCA(A) | 105 | 1.69 | GAA(E) | 168 | 2 | GGA(G) | 8 | 0.12 |
|  | GUG(V) | 3 | 0.03 | GCG(A) | 5 | 0.08 | GAG(E) | 0 | 0 | GGG(G) | 0 | 0 |
|  | **U** | | | **C** | | | **A** | | | **G** | | |

**Table S2Be**. Codon usage in *Blastocystis* sp. ST3 DMP/IH:478 (HQ909888)

^†^Including in-frame stop codon in *orf160*.

|  | **Codon** | **Count** | **RSCU** | **Codon** | **Count** | **RSCU** | **Codon** | **Count** | **RSCU** | **Codon** | **Count** | **RSCU** |
| --- | --- | --- | --- | --- | --- | --- | --- | --- | --- | --- | --- | --- |
| **U** | UUU(F) | 591 | 1.92 | UCU(S) | 128 | 1.66 | UAU(Y) | 468 | 1.93 | UGU(C) | 114 | 1.93 |
|  | UUC(F) | 25 | 0.08 | UCC(S) | 11 | 0.14 | UAC(Y) | 16 | 0.07 | UGC(C) | 4 | 0.07 |
|  | UUA(L) | 765 | 4.93 | UCA(S) | 162 | 2.1 | UAA(*) | 26 | 2.89 | UGA(*) | 1 | 0.11 |
|  | UUG(L) | 46 | 0.3 | UCG(S) | 12 | 0.16 | UAG(*) | 0 | 0 | UGG(W) | 41 | 1 |
| **C** | CUU(L) | 92 | 0.59 | CCU(P) | 67 | 1.62 | CAU(H) | 120 | 1.8 | CGU(R) | 113 | 3.57 |
|  | CUC(L) | 8 | 0.05 | CCC(P) | 2 | 0.05 | CAC(H) | 13 | 0.2 | CGC(R) | 10 | 0.32 |
|  | CUA(L) | 17 | 0.11 | CCA(P) | 91 | 2.21 | CAA(Q) | 249 | 1.95 | CGA(R) | 40 | 1.26 |
|  | CUG(L) | 3 | 0.02 | CCG(P) | 5 | 0.12 | CAG(Q) | 7 | 0.05 | CGG(R) | 2 | 0.06 |
| **A** | AUU(I) | 628 | 2.2 | ACU(T) | 181 | 1.65 | AAU(N) | 576 | 1.91 | AGU(S) | 136 | 1.76 |
|  | AUC(I) | 30 | 0.11 | ACC(T) | 22 | 0.2 | AAC(N) | 27 | 0.09 | AGC(S) | 14 | 0.18 |
|  | AUA(I) | 199 | 0.7 | ACA(T) | 222 | 2.02 | AAA(K) | 580 | 1.94 | AGA(R) | 24 | 0.76 |
|  | AUG(M) | 132 | 1 | ACG(T) | 15 | 0.14 | AAG(K) | 18 | 0.06 | AGG(R) | 1 | 0.03 |
| **G** | GUU(V) | 181 | 2.47 | GCU(A) | 111 | 1.88 | GAU(D) | 172 | 1.95 | GGU(G) | 161 | 2.48 |
|  | GUC(V) | 7 | 0.1 | GCC(A) | 7 | 0.12 | GAC(D) | 4 | 0.05 | GGC(G) | 5 | 0.08 |
|  | GUA(V) | 92 | 1.26 | GCA(A) | 114 | 1.93 | GAA(E) | 158 | 1.98 | GGA(G) | 90 | 1.38 |
|  | GUG(V) | 13 | 0.18 | GCG(A) | 4 | 0.07 | GAG(E) | 2 | 0.03 | GGG(G) | 4 | 0.06 |
|  | **U** | | | **C** | | | **A** | | | **G** | | |

**Table S2Bf.** Codon usage in *Blastocystis* sp. ST4 DMP/10-212 (KU900236)

|  | **Codon** | **Count** | **RSCU** | **Codon** | **Count** | **RSCU** | **Codon** | **Count** | **RSCU** | **Codon** | **Count** | **RSCU** |
| --- | --- | --- | --- | --- | --- | --- | --- | --- | --- | --- | --- | --- |
| **U** | UUU(F) | 577 | 1.89 | UCU(S) | 109 | 1.44 | UAU(Y) | 461 | 1.9 | UGU(C) | 104 | 1.89 |
|  | UUC(F) | 35 | 0.11 | UCC(S) | 11 | 0.15 | UAC(Y) | 25 | 0.1 | UGC(C) | 6 | 0.11 |
|  | UUA(L) | 769 | 4.89 | UCA(S) | 161 | 2.13 | UAA(*) | 24 | 2.67 | UGA(*) | 1 | 0.11 |
|  | UUG(L) | 66 | 0.42 | UCG(S) | 29 | 0.38 | UAG(*) | 2 | 0.22 | UGG(W) | 42 | 1 |
| **C** | CUU(L) | 86 | 0.55 | CCU(P) | 63 | 1.54 | CAU(H) | 118 | 1.89 | CGU(R) | 108 | 3.38 |
|  | CUC(L) | 6 | 0.04 | CCC(P) | 6 | 0.15 | CAC(H) | 7 | 0.11 | CGC(R) | 9 | 0.28 |
|  | CUA(L) | 13 | 0.08 | CCA(P) | 90 | 2.2 | CAA(Q) | 244 | 1.83 | CGA(R) | 52 | 1.63 |
|  | CUG(L) | 4 | 0.03 | CCG(P) | 5 | 0.12 | CAG(Q) | 23 | 0.17 | CGG(R) | 1 | 0.03 |
| **A** | AUU(I) | 607 | 2.19 | ACU(T) | 164 | 1.38 | AAU(N) | 595 | 1.92 | AGU(S) | 135 | 1.79 |
|  | AUC(I) | 30 | 0.11 | ACC(T) | 22 | 0.19 | AAC(N) | 26 | 0.08 | AGC(S) | 8 | 0.11 |
|  | AUA(I) | 193 | 0.7 | ACA(T) | 273 | 2.3 | AAA(K) | 570 | 1.95 | AGA(R) | 21 | 0.66 |
|  | AUG(M) | 122 | 1 | ACG(T) | 15 | 0.13 | AAG(K) | 15 | 0.05 | AGG(R) | 1 | 0.03 |
| **G** | GUU(V) | 172 | 2.42 | GCU(A) | 100 | 1.82 | GAU(D) | 175 | 1.93 | GGU(G) | 163 | 2.49 |
|  | GUC(V) | 14 | 0.2 | GCC(A) | 10 | 0.18 | GAC(D) | 6 | 0.07 | GGC(G) | 7 | 0.11 |
|  | GUA(V) | 82 | 1.15 | GCA(A) | 105 | 1.91 | GAA(E) | 151 | 2 | GGA(G) | 87 | 1.33 |
|  | GUG(V) | 16 | 0.23 | GCG(A) | 5 | 0.09 | GAG(E) | 0 | 0 | GGG(G) | 5 | 0.08 |
|  | **U** | | | **C** | | | **A** | | | **G** | | |

**Table S2Bg**. Codon usage in *Blastocystis* sp. ST4 DMP/02-328 (EF494739)

|  | **Codon** | **Count** | **RSCU** | **Codon** | **Count** | **RSCU** | **Codon** | **Count** | **RSCU** | **Codon** | **Count** | **RSCU** |
| --- | --- | --- | --- | --- | --- | --- | --- | --- | --- | --- | --- | --- |
| **U** | UUU(F) | 585 | 1.87 | UCU(S) | 181 | 2.51 | UAU(Y) | 544 | 1.92 | UGU(C) | 89 | 1.87 |
|  | UUC(F) | 41 | 0.13 | UCC(S) | 5 | 0.07 | UAC(Y) | 22 | 0.08 | UGC(C) | 6 | 0.13 |
|  | UUA(L) | 823 | 5.23 | UCA(S) | 93 | 1.29 | UAA(*) | 25 | 2.78 | UGA(*) | 1 | 0.11 |
|  | UUG(L) | 64 | 0.41 | UCG(S) | 2 | 0.03 | UAG(*)^†^ | 1 | 0.11 | UGG(W) | 41 | 1 |
| **C** | CUU(L) | 44 | 0.28 | CCU(P) | 77 | 2.08 | CAU(H) | 96 | 1.96 | CGU(R) | 77 | 2.08 |
|  | CUC(L) | 0 | 0 | CCC(P) | 0 | 0 | CAC(H) | 2 | 0.04 | CGC(R) | 2 | 0.05 |
|  | CUA(L) | 12 | 0.08 | CCA(P) | 69 | 1.86 | CAA(Q) | 138 | 2 | CGA(R) | 22 | 0.59 |
|  | CUG(L) | 1 | 0.01 | CCG(P) | 2 | 0.05 | CAG(Q) | 0 | 0 | CGG(R) | 0 | 0 |
| **A** | AUU(I) | 522 | 1.68 | ACU(T) | 168 | 2.23 | AAU(N) | 813 | 1.92 | AGU(S) | 146 | 2.03 |
|  | AUC(I) | 13 | 0.04 | ACC(T) | 7 | 0.09 | AAC(N) | 32 | 0.08 | AGC(S) | 5 | 0.07 |
|  | AUA(I) | 398 | 1.28 | ACA(T) | 125 | 1.66 | AAA(K) | 717 | 1.91 | AGA(R) | 120 | 3.24 |
|  | AUG(M) | 126 | 1 | ACG(T) | 1 | 0.01 | AAG(K) | 32 | 0.09 | AGG(R) | 1 | 0.03 |
| **G** | GUU(V) | 170 | 2.3 | GCU(A) | 121 | 2.38 | GAU(D) | 213 | 1.97 | GGU(G) | 208 | 3.28 |
|  | GUC(V) | 6 | 0.08 | GCC(A) | 3 | 0.06 | GAC(D) | 3 | 0.03 | GGC(G) | 4 | 0.06 |
|  | GUA(V) | 113 | 1.53 | GCA(A) | 77 | 1.52 | GAA(E) | 183 | 1.99 | GGA(G) | 42 | 0.66 |
|  | GUG(V) | 7 | 0.09 | GCG(A) | 2 | 0.04 | GAG(E) | 1 | 0.01 | GGG(G) | 0 | 0 |
|  | **U** | | | **C** | | | **A** | | | **G** | | |

**Table S2Bh**. Codon usage in *Blastocystis* sp. ST6 SSI:754 (KU900237)

^†^Including in-frame stop codon in *orf160*.

|  | **Codon** | **Count** | **RSCU** | **Codon** | **Count** | **RSCU** | **Codon** | **Count** | **RSCU** | **Codon** | **Count** | **RSCU** |
| --- | --- | --- | --- | --- | --- | --- | --- | --- | --- | --- | --- | --- |
| **U** | UUU(F) | 478 | 1.68 | UCU(S) | 243 | 3.11 | UAU(Y) | 556 | 1.92 | UGU(C) | 85 | 1.81 |
|  | UUC(F) | 90 | 0.32 | UCC(S) | 9 | 0.12 | UAC(Y) | 22 | 0.08 | UGC(C) | 9 | 0.19 |
|  | UUA(L) | 809 | 5.38 | UCA(S) | 67 | 0.86 | UAA(*) | 24 | 1.71 | UGA(W) | 0 | 0 |
|  | UUG(L) | 32 | 0.21 | UCG(S) | 2 | 0.03 | UAG(*)^†^ | 4 | 0.29 | UGG(W) | 41 | 2 |
| **C** | CUU(L) | 53 | 0.35 | CCU(P) | 98 | 2.55 | CAU(H) | 107 | 1.86 | CGU(R) | 100 | 2.43 |
|  | CUC(L) | 4 | 0.03 | CCC(P) | 1 | 0.03 | CAC(H) | 8 | 0.14 | CGC(R) | 0 | 0 |
|  | CUA(L) | 3 | 0.02 | CCA(P) | 55 | 1.43 | CAA(Q) | 155 | 2 | CGA(R) | 5 | 0.12 |
|  | CUG(L) | 1 | 0.01 | CCG(P) | 0 | 0 | CAG(Q) | 0 | 0 | CGG(R) | 0 | 0 |
| **A** | AUU(I) | 576 | 1.99 | ACU(T) | 223 | 2.71 | AAU(N) | 756 | 1.9 | AGU(S) | 139 | 1.78 |
|  | AUC(I) | 31 | 0.11 | ACC(T) | 10 | 0.12 | AAC(N) | 41 | 0.1 | AGC(S) | 9 | 0.12 |
|  | AUA(I) | 263 | 0.91 | ACA(T) | 96 | 1.17 | AAA(K) | 762 | 2 | AGA(R) | 142 | 3.45 |
|  | AUG(M) | 138 | 1 | ACG(T) | 0 | 0 | AAG(K) | 1 | 0 | AGG(R) | 0 | 0 |
| **G** | GUU(V) | 207 | 2.22 | GCU(A) | 148 | 2.61 | GAU(D) | 214 | 1.98 | GGU(G) | 242 | 3.57 |
|  | GUC(V) | 6 | 0.06 | GCC(A) | 3 | 0.05 | GAC(D) | 2 | 0.02 | GGC(G) | 5 | 0.07 |
|  | GUA(V) | 155 | 1.66 | GCA(A) | 75 | 1.32 | GAA(E) | 187 | 2 | GGA(G) | 23 | 0.34 |
|  | GUG(V) | 5 | 0.05 | GCG(A) | 1 | 0.02 | GAG(E) | 0 | 0 | GGG(G) | 1 | 0.01 |
|  | **U** | | | **C** | | | **A** | | | **G** | | |

**Table S2Bi**. Codon usage in *Blastocystis* sp. ST7 B (CU914152)

^†^including single in-frame stop codon in *orf160*

|  | **Codon** | **Count** | **RSCU** | **Codon** | **Count** | **RSCU** | **Codon** | **Count** | **RSCU** | **Codon** | **Count** | **RSCU** |
| --- | --- | --- | --- | --- | --- | --- | --- | --- | --- | --- | --- | --- |
| **U** | UUU(F) | 601 | 1.89 | UCU(S) | 78 | 1.13 | UAU(Y) | 455 | 1.94 | UGU(C) | 106 | 1.89 |
|  | UUC(F) | 34 | 0.11 | UCC(S) | 19 | 0.27 | UAC(Y) | 15 | 0.06 | UGC(C) | 6 | 0.11 |
|  | UUA(L) | 711 | 4.52 | UCA(S) | 149 | 2.15 | UAA(*) | 25 | 2.59 | UGA(*)^†^ | 1 | 0.1 |
|  | UUG(L) | 89 | 0.57 | UCG(S) | 37 | 0.53 | UAG(*)^†^ | 3 | 0.31 | UGG(W) | 45 | 1 |
| **C** | CUU(L) | 98 | 0.62 | CCU(P) | 47 | 1.15 | CAU(H) | 123 | 1.81 | CGU(R) | 95 | 2.52 |
|  | CUC(L) | 5 | 0.03 | CCC(P) | 1 | 0.02 | CAC(H) | 13 | 0.19 | CGC(R) | 10 | 0.27 |
|  | CUA(L) | 35 | 0.22 | CCA(P) | 109 | 2.67 | CAA(Q) | 292 | 1.9 | CGA(R) | 82 | 2.18 |
|  | CUG(L) | 6 | 0.04 | CCG(P) | 6 | 0.15 | CAG(Q) | 15 | 0.1 | CGG(R) | 12 | 0.32 |
| **A** | AUU(I) | 627 | 2.23 | ACU(T) | 119 | 1.05 | AAU(N) | 570 | 1.88 | AGU(S) | 126 | 1.82 |
|  | AUC(I) | 37 | 0.13 | ACC(T) | 26 | 0.23 | AAC(N) | 38 | 0.13 | AGC(S) | 7 | 0.1 |
|  | AUA(I) | 181 | 0.64 | ACA(T) | 279 | 2.46 | AAA(K) | 552 | 1.94 | AGA(R) | 25 | 0.66 |
|  | AUG(M) | 131 | 1 | ACG(T) | 30 | 0.26 | AAG(K) | 18 | 0.06 | AGG(R) | 2 | 0.05 |
| **G** | GUU(V) | 186 | 2.58 | GCU(A) | 71 | 1.19 | GAU(D) | 176 | 1.92 | GGU(G) | 128 | 1.96 |
|  | GUC(V) | 16 | 0.22 | GCC(A) | 7 | 0.12 | GAC(D) | 7 | 0.08 | GGC(G) | 9 | 0.14 |
|  | GUA(V) | 73 | 1.01 | GCA(A) | 147 | 2.47 | GAA(E) | 164 | 1.96 | GGA(G) | 113 | 1.73 |
|  | GUG(V) | 13 | 0.18 | GCG(A) | 13 | 0.22 | GAG(E) | 3 | 0.04 | GGG(G) | 11 | 0.17 |
|  | **U** | | | **C** | | | **A** | | | **G** | | |

**Table S2Bj**. Codon usage in *Blastocystis* sp. ST8 DMP/08-128 (KU900238)

^†^Including two in-frame stop codons in *orf160*.

|  | **Codon** | **Count** | **RSCU** | **Codon** | **Count** | **RSCU** | **Codon** | **Count** | **RSCU** | **Codon** | **Count** | **RSCU** |
| --- | --- | --- | --- | --- | --- | --- | --- | --- | --- | --- | --- | --- |
| **U** | UUU(F) | 604 | 1.9 | UCU(S) | 183 | 2.57 | UAU(Y) | 548 | 1.93 | UGU(C) | 87 | 1.87 |
|  | UUC(F) | 31 | 0.1 | UCC(S) | 4 | 0.06 | UAC(Y) | 21 | 0.07 | UGC(C) | 6 | 0.13 |
|  | UUA(L) | 808 | 5.16 | UCA(S) | 91 | 1.28 | UAA(*) | 24 | 2.57 | UGA(*) | 1 | 0.11 |
|  | UUG(L) | 65 | 0.41 | UCG(S) | 3 | 0.04 | UAG(*)^†^ | 3 | 0.32 | UGG(W) | 41 | 1 |
| **C** | CUU(L) | 41 | 0.26 | CCU(P) | 76 | 2.03 | CAU(H) | 94 | 1.92 | CGU(R) | 79 | 2.31 |
|  | CUC(L) | 3 | 0.02 | CCC(P) | 2 | 0.05 | CAC(H) | 4 | 0.08 | CGC(R) | 1 | 0.03 |
|  | CUA(L) | 22 | 0.14 | CCA(P) | 72 | 1.92 | CAA(Q) | 142 | 1.97 | CGA(R) | 20 | 0.59 |
|  | CUG(L) | 1 | 0.01 | CCG(P) | 0 | 0 | CAG(Q) | 2 | 0.03 | CGG(R) | 0 | 0 |
| **A** | AUU(I) | 528 | 1.68 | ACU(T) | 167 | 2.21 | AAU(N) | 818 | 1.92 | AGU(S) | 139 | 1.95 |
|  | AUC(I) | 10 | 0.03 | ACC(T) | 8 | 0.11 | AAC(N) | 32 | 0.08 | AGC(S) | 8 | 0.11 |
|  | AUA(I) | 405 | 1.29 | ACA(T) | 126 | 1.67 | AAA(K) | 733 | 1.93 | AGA(R) | 105 | 3.07 |
|  | AUG(M) | 126 | 1 | ACG(T) | 1 | 0.01 | AAG(K) | 28 | 0.07 | AGG(R) | 0 | 0 |
| **G** | GUU(V) | 175 | 2.36 | GCU(A) | 124 | 2.46 | GAU(D) | 210 | 1.99 | GGU(G) | 209 | 3.28 |
|  | GUC(V) | 4 | 0.05 | GCC(A) | 2 | 0.04 | GAC(D) | 1 | 0.01 | GGC(G) | 2 | 0.03 |
|  | GUA(V) | 113 | 1.52 | GCA(A) | 74 | 1.47 | GAA(E) | 178 | 1.95 | GGA(G) | 43 | 0.67 |
|  | GUG(V) | 5 | 0.07 | GCG(A) | 2 | 0.04 | GAG(E) | 5 | 0.05 | GGG(G) | 1 | 0.02 |
|  | **U** | | | **C** | | | **A** | | | **G** | | |

**Table S2Bk**. Codon usage in *Blastocystis* sp. ST9 F5323 (KU900239).

^†^Including in-frame stop codon in *orf160*.
