# Supplementary material for: Blastocystis Mitochondrial Genomes Appear to Show Multiple Independent Gains and Losses of Start and Stop Codons
Source: Genome Biol Evol. 2016 Nov 9;8(11):3340–50. doi: 10.1093/gbe/evw255 (PMC5203790; doi:10.1093/gbe/evw255)
Supplement: Supplementary Data [file supp_evw255_suppl_data.zip › Supplementary_Table_S3.docx]

**Supplementary Table S3**:

Termination codon usage and reassignment

1. Termination codon usage in the 27 protein genes in the MRO genomes of *Blastocystis*.

| *Blastocystis* sp. | GenBank  Accession | UGA | UAA | UAG |
| --- | --- | --- | --- | --- |
| ST1 NandII | EF494740 | 0 | 26 | 1 (*rps19*) |
| ST2 Flemming | KU900235 | 0 | 27 | 0 |
| ST3 DMP/IH:478 | HQ909888 | 0 | 26 | 1 (*rpl5*) |
| ST3 DMP/08-326 | HQ909886 | 0 | 26 | 1 (*rpl5*) |
| ST3 DMP/08-1043 | HQ909887 | 0 | 25 | 2 (*rps8*, *rpl5*) |
| ST4 DMP/10-212 | KU900236 | 1 (*rps19*) | 26 | 0 |
| ST4 DMP/02-328 | EF494739 | 1 (*rps19*) | 24 | 2 (*rps14*, *nad4l*) |
| ST6 SSI:754 | KU900237 | 1 (*rps19*) | 25 | 1 (*nad6*) |
| ST7 B | CU914152 | 0 | 24 | 3 (*nad4*, *rps11*, *nad9*) |
| ST8 DMP/ 08-128 | KU900238 | 0 | 26 | 1 (*rps2*) |
| ST9 F5323 | KU900239 | 1 (*rps19*) | 24 | 2 (*nad6*, *rps12*) |

B. Codon usage across stramenopile MRO genomes relevant to reassignments of stop codon UGA and UAG

| Class |  | GenBank  Accession | UGA is - | tRNA-Trp anticodon | tRNA-Leu anticodon | UGA | UAA | UAG |
| --- | --- | --- | --- | --- | --- | --- | --- | --- |
| *Blastocystis* | *Blastocystis* sp. ST1 NandII | EF494740 | Not used | CCA | UAA | 0 | 26 | 1 (term)^2^  2 (stop)^3^ |
|  | *Blastocystis* sp. ST2 Flemming | KU900235 | Not used | CCA | UAA | 0 | 27 | 2 (stop)^3^ |
|  | *Blastocystis* sp. ST3 DMP/IH478 | HQ909888 | Not used | CCA | UAA | 0 | 26 | 1 (stop)^2^ |
|  | *Blastocystis* sp. ST3  DMP/08-326 | HQ909886 | Not used | CCA | UAA | 0 | 26 | 1 (term)^2^  1 (stop)^3^ |
|  | *Blastocystis* sp. ST3  DMP/08-1043 | HQ909887 | Not used | CCA | UAA | 0 | 25 | 2 (term)^2^  1 (stop)^3^ |
|  | *Blastocystis* sp. ST4  DMP/10-212 | KU900236 | term^2^ | CCA | UAA | 1 | 26 | 0 |
|  | *Blastocystis* sp. ST4  DMP/02-328 | EF494739 | term | CCA | UAA | 1 | 24 | 2 |
|  | *Blastocystis* sp. ST6 SSI:754 | KU900237 | term | CAA | UAA | 1 | 24 | 2 (term)^2^  1 (stop)^3^ |
|  | *Blastocystis* sp. ST7 B | CU914152 | Not used | CCA | UAA | 0 | 24 | 3 (term)  1 (stop) |
|  | *Blastocystis* sp. ST8  DMP/08-128 | KU900238 | term | CCA | UAA | 1 (stop)^3^ | 26 | 1 (term)  1 (stop) |
|  | *Blastocystis* sp. ST9 F5323 | KU900239 | term | CCA | UAA | 1 | 24 | 2 (term)  1 (stop) |
| Biscosoecida | *Caferteria roenbergensis*^1^ | NC_000946 | Trp | TCA | UAA | 190 | 32 | 2 |
| Peronosporomycetes | *Phytophthora infestans*^1^ | NC_002387 | term | CCA | UAG & UAA | 1 | 39 | 0 |
|  | *Phytophthora sojae* | NC_009385 | term | CCA | UAG & UAA | 2 | 43 | 2 |
|  | *Phytophthora ramorum* | NC_009384 | term | CCA | UAG & UAA | 1 | 42 | 0 |
|  | *Phytophthora andina* | [NC_015619](http://www.ncbi.nlm.nih.gov/nuccore/NC_015619.1) | term | CCA | UAG & UAA | 1 | 39 | 0 |
|  | *Phytophthora mirabilis* | [NC_015606](http://www.ncbi.nlm.nih.gov/nuccore/NC_015606.1) | term | CCA | UAG & UAA | 1 | 39 | 0 |
|  | *Phytophthora phaseoli* | NC_015616 | term | CCA | UAA & UAG | 1 | 39 | 0 |
|  | *Phytophthora ipomoeae* | [NC_015622](http://www.ncbi.nlm.nih.gov/nuccore/NC_015622.1) | term | CCA | UAA & UAG | 1 | 39 | 0 |
|  | *Saprolegnia ferax*^1^ | [NC_005984](http://www.ncbi.nlm.nih.gov/nuccore/NC_005984.1) | Not used | CCA | UAA & UAG | 0 | 42 | 1 |
|  | *Thraustotheca clavata* | [NC_022179](http://www.ncbi.nlm.nih.gov/nuccore/NC_022179.1) | term | CCA | UAG, UAG &  UAA | 0 | 39 | 2 |
|  | *Achlya hypogyna* | [NC_022178](http://www.ncbi.nlm.nih.gov/nuccore/NC_022178.1) | Not used | CCA | UAA & UAG | 0 | 43 | 0 |
|  | *Pythium ultimum* | NC_014280 | Not used | CCA | UAA & UAG | 0 | 65 | 2 |
| Synurales | *Chrysodidymus synuroides*^1^ | [NC_002174](http://www.ncbi.nlm.nih.gov/nuccore/NC_002174.1) | Not used | CCA | UAA & UAG | 0 | 34 | 3 |
| Chrysophyceae | *Ochramonas danica*^1^ | [NC_002571](http://www.ncbi.nlm.nih.gov/nuccore/NC_002571.1) | Not used | CCA | UAA & UAG | 0 | 30 | 14 |
| Opalinata | *Proteromonas lacertae* | NC_014338 | Not used | CAA | UAG & UAA | 0 | 54 | 0 |
| Diatomea | *Ulnaria acus* | [NC_013710](http://www.ncbi.nlm.nih.gov/nuccore/NC_013710.1) | term | CCA | UAG | 1 | 24 | 10 |
|  | *Phaeodactylum tricornutum* | NC_016739 | Not used | CCA | UAA & UAG | 0 | 28 | 5 |
|  | *Thalassiosira pseudonana*^1^ | NC_007405 | Trp | UCA & CCA | UAG & UAA | 62 | 32 | 3 |
| Eustigmatales | *Nannochloropsis gaditana* | NC_020015 | Not used | CCA | UAG, CAA &  UAA | 0 | 30 | 3 |
|  | *Nannochloropsis granulata* | [NC_022254.1](http://www.ncbi.nlm.nih.gov/nuccore/NC_022254.1) | Not used | CCA | UAG, CAA &  UAA | 0 | 33 | 2 |
|  | *Nannochloropsis limnetica* | [NC_022256.1](http://www.ncbi.nlm.nih.gov/nuccore/NC_022256.1) | Not used | CCA | UAG, CAA &  UAA | 0 | 33 | 2 |
|  | *Nannochloropsis oceanica* | [NC_022258](http://www.ncbi.nlm.nih.gov/nuccore/NC_022258.1) | Not used | CCA | UAG, CAA &  UAA | 0 | 31 | 4 |
|  | *Nannochloropsis oculata* | [NC_022257](http://www.ncbi.nlm.nih.gov/nuccore/NC_022257.1) | term | CCA | UAG, CAA &  UAA | 1 | 33 | 1 |
|  | *Nannochloropsis salina* | [NC_022255](http://www.ncbi.nlm.nih.gov/nuccore/NC_022255.1) | Not used | CCA | UAG, CAA &  UAA | 0 | 33 | 3 |
| Raphidophyceae | *Heterosigma akashiwo* | [NC_016738](http://www.ncbi.nlm.nih.gov/nuccore/NC_016738.1) | term | CCA | UAA & UAG | 1 | 39 | 1 |
|  | *Chattonella marina* | [NC_013837](http://www.ncbi.nlm.nih.gov/nuccore/NC_013837.1) | term | CCA | UAG & UAA | 1 | 36 | 4 |
| Phaeophyceae | *Dictyota dichotoma* | NC_007685 | term | CCA | UAA, UAG  & CAA | 8 | 24 | 6 |
|  | *Fucus vesiculosus* | NC_007683 | term | CCA | UAA, UAG  & CAA | 2 | 31 | 5 |
|  | *Desmarestia viridis* | NC_007684 | term | CCA | UAA, UAG  & CAA | 2 | 31 | 6 |
|  | *Saccharina angustata* | NC_013473 | term | CCA | UAG & CAA | 4 | 29 | 5 |
|  | *Saccharina coriacea* | NC_013475 | term | CCA | UAG & CAA | 4 | 27 | 7 |
|  | *Saccharina religiosa* | NC_013477 | term | CCA | UAG & CAA | 4 | 26 | 8 |
|  | *Saccharina longipedalis* | NC_013484 | term | CCA | UAG & CAA | 4 | 26 | 8 |
|  | *Saccharina japonica* x *latissima* | NC_015669 | term | CCA | UAG & CAA | 4 | 26 | 8 |
|  | *Saccharina ochotensis* | NC_013478 | term | CCA | UAG & CAA | 4 | 26 | 8 |
|  | *Saccharina japonica* | NC_013476 | term | CCA | UAG & CAA | 4 | 26 | 8 |
|  | *Saccharina diabolica* | NC_013482 | term | CCA | UAG & CAA | 4 | 26 | 8 |
|  | [*Costaria costata*](http://www.ncbi.nlm.nih.gov/Taxonomy/Browser/wwwtax.cgi?lvl=0&id=2872) | NC_023506 | term | CAA | UAA, UAG & CAA | 4 | 27 | 7 |
|  | [*Undaria pinnatifida*](http://www.ncbi.nlm.nih.gov/Taxonomy/Browser/wwwtax.cgi?lvl=0&id=74381) | NC_023354 | term | CCA | UAA, UAG & CAA | 3 | 30 | 5 |
|  | *Laminaria digitata*^1^ | NC_004024 | term | CCA | UAA, UAG  & CAA | 4 | 29 | 6 |
|  | *Pylaiella littoralis*^1^ | NC_003055 | term | CCA | UAA & UAG | 7 | 38 | 7 |

^1^Sengupta S, Yang X, Higgs PG. 2007. The mechanisms of codon reassignments in mitochondrial genetic codes. J Mol Evol. **64**: 662-688.

^2^Termination codon.

^3^Stop codon (in-frame) found in homologues of *orf160*.
