# Supplementary material for: Blastocystis Mitochondrial Genomes Appear to Show Multiple Independent Gains and Losses of Start and Stop Codons
Source: Genome Biol Evol. 2016 Nov 9;8(11):3340–50. doi: 10.1093/gbe/evw255 (PMC5203790; doi:10.1093/gbe/evw255)
Supplement: Supplementary Data [file supp_evw255_suppl_data.zip › Supplementary_Table_S4.docx]

Supplementary Table S4. Percent identity of MRO genes and proteins across subtypes

| *nad 3* | **Subtype 1 NandII** | **Subtype 2 Flemming** | **Subtype 3 DMP/08-326** | **Subtype 3 DMP/ 08-1043** | **Subtype 3 IH:478** | **Subtype 4 DMP/02-328** | **Subtype 4 DMP/10-212** | **Subtype 6 SSI 754** | **Subtype 7 S7-B** | **Subtype 8 DMP/08-128** | **Subtype 9 F5323** |
| --- | --- | --- | --- | --- | --- | --- | --- | --- | --- | --- | --- |
| **Subtype 1 NandII** |  | 89.2 | 86.9 | 86.4 | 86.9 | 85.8 | 84.7 | 85.8 | 88.9 | 82.5 | 86.7 |
| **Subtype 2 Flemming** | 87.4 |  | 88.3 | 87.8 | 88.3 | 88.3 | 87.2 | 84.7 | 88.9 | 84.4 | 85.6 |
| **Subtype 3 DMP/08-326** | 85.7 | 86.6 |  | 98.6 | 100 | 89.2 | 87.5 | 85.3 | 86.1 | 87.2 | 86.4 |
| **Subtype 3 DMP/ 08-1043** | 85.7 | 86.6 | 98.3 |  | 98.6 | 89.2 | 86.9 | 85.3 | 86.1 | 87.2 | 86.4 |
| **Subtype 3 IH:478** | 85.7 | 86.6 | 100 | 98.3 |  | 89.2 | 87.5 | 85.3 | 86.1 | 87.2 | 86.4 |
| **Subtype 4 DMP/02-328** | 86.6 | 89.1 | 90.8 | 90.8 | 90.8 |  | 92.5 | 85 | 86.4 | 90 | 85 |
| **Subtype 4 DMP/10-212** | 85.7 | 87.4 | 90.8 | 90.8 | 90.8 | 95 |  | 85 | 86.1 | 89.4 | 85 |
| **Subtype 6 SSI 754** | 85.7 | 82.4 | 84.9 | 84.9 | 84.9 | 89.1 | 85.7 |  | 85.6 | 84.2 | 97.8 |
| **Subtype 7 S7-B** | 88.2 | 85.7 | 84.9 | 84.9 | 84.9 | 86.6 | 84 | 84.9 |  | 83.3 | 86.4 |
| **Subtype 8 DMP/08-128** | 85.7 | 87.4 | 89.1 | 89.1 | 89.1 | 95.8 | 94.1 | 86.6 | 83.2 |  | 84.7 |
| **Subtype 9 F5323** | 85.7 | 82.4 | 85.7 | 85.7 | 85.7 | 89.1 | 85.7 | 99.2 | 84.9 | 86.6 |  |

Upper diagonal values are for nucleotide, lower diagonal values correspond to amino acids

| *nad 6* | **Subtype 1 NandII** | **Subtype 2 Flemming** | **Subtype 3 DMP/08-326** | **Subtype 3 DMP/ 08-1043** | **Subtype 3 IH:478** | **Subtype 4 DMP/02-328** | **Subtype 4 DMP/10-212** | **Subtype 6 SSI 754** | **Subtype 7 S7-B** | **Subtype 8 DMP/08-128** | **Subtype 9 F5323** |
| --- | --- | --- | --- | --- | --- | --- | --- | --- | --- | --- | --- |
| **Subtype 1 NandII** |  | 91.5 | 85.1 | 85.1 | 85 | 85.1 | 85.1 | 89.2 | 88.2 | 85 | 88.5 |
| **Subtype 2 Flemming** | 95.4 |  | 86.3 | 86 | 86.2 | 84.1 | 84.8 | 88.5 | 88.5 | 84.8 | 87.9 |
| **Subtype 3 DMP/08-326** | 85.1 | 87.6 |  | 98.3 | 99.8 | 81.9 | 83.9 | 84.6 | 86 | 83.9 | 85.3 |
| **Subtype 3 DMP/ 08-1043** | 85.1 | 87.6 | 99.5 |  | 98.1 | 82.6 | 83.8 | 84.8 | 85.1 | 83.6 | 85.5 |
| **Subtype 3 IH:478** | 85.1 | 87.6 | 100 | 99.5 |  | 81.9 | 83.8 | 84.4 | 85.8 | 83.8 | 85.1 |
| **Subtype 4 DMP/02-328** | 85.6 | 85.6 | 84 | 84.5 | 84 |  | 91.6 | 83.8 | 85 | 87.4 | 83.9 |
| **Subtype 4 DMP/10-212** | 83 | 83 | 84 | 83.5 | 84 | 91.8 |  | 85.1 | 84.3 | 86.2 | 85.3 |
| **Subtype 6 SSI 754** | 90.7 | 87.1 | 83.5 | 83.5 | 83.5 | 84.5 | 81.4 |  | 90.9 | 85.1 | 96.4 |
| **Subtype 7 S7-B** | 89.2 | 88.1 | 85.1 | 84.5 | 85.1 | 85.1 | 82 | 92.8 |  | 84.1 | 90.4 |
| **Subtype 8 DMP/08-128** | 84.5 | 85.1 | 84 | 84 | 84 | 88.7 | 85.1 | 81.4 | 80.9 |  | 85.3 |
| **Subtype 9 F5323** | 89.2 | 86.6 | 83.5 | 83.5 | 83.5 | 84 | 82 | 97.9 | 90.7 | 80.9 |  |

Upper diagonal values are for nucleotide, lower diagonal values correspond to amino acids

| *Rps13* | **Subtype 1 NandII** | **Subtype 2 Flemming** | **Subtype 3 DMP/08-326** | **Subtype 3 DMP/ 08-1043** | **Subtype 3 IH:478** | **Subtype 4 DMP/02-328** | **Subtype 4 DMP/10-212** | **Subtype 6 SSI 754** | **Subtype 7 S7-B** | **Subtype 8 DMP/08-128** | **Subtype 9 F5323** |
| --- | --- | --- | --- | --- | --- | --- | --- | --- | --- | --- | --- |
| **Subtype 1 NandII** |  | 88.2 | 78.5 | 80.8 | 78.5 | 70.8 | 72.6 | 80.8 | 79.6 | 77 | 79.9 |
| **Subtype 2 Flemming** | 88.4 |  | 82 | 82.6 | 82 | 72.6 | 74 | 82.3 | 77.9 | 75.8 | 81.1 |
| **Subtype 3 DMP/08-326** | 76.8 | 80.4 |  | 95 | 100 | 73.5 | 73.2 | 77.9 | 75.2 | 73.5 | 77.9 |
| **Subtype 3DMP/ 08-1043** | 76.8 | 80.4 | 99.1 |  | 95 | 74.9 | 74 | 80.2 | 76.4 | 75.5 | 79.6 |
| **Subtype 3 IH:478** | 76.8 | 80.4 | 100 | 99.1 |  | 73.5 | 73.2 | 77.9 | 75.2 | 73.5 | 77.9 |
| **Subtype 4 DMP/02-328** | 64.3 | 67.9 | 67.9 | 67.9 | 67.9 |  | 89.1 | 70.2 | 70.5 | 77.3 | 69 |
| **Subtype 4 DMP/10-212** | 67 | 70.5 | 67 | 67 | 67 | 92 |  | 71.1 | 68.4 | 76.7 | 69 |
| **Subtype 6 SSI 754** | 72.3 | 73.2 | 74.1 | 74.1 | 74.1 | 60.7 | 59.8 |  | 84.1 | 73.2 | 96.5 |
| **Subtype 7 S7-B** | 69.6 | 67 | 65.2 | 65.2 | 65.2 | 58.9 | 58 | 78.6 |  | 71.7 | 83.8 |
| **Subtype 8 DMP/08-128** | 68.8 | 68.8 | 68.8 | 68.8 | 68.8 | 70.5 | 71.4 | 63.4 | 54.5 |  | 71.4 |
| **Subtype 9 F5323** | 73.2 | 74.1 | 74.1 | 74.1 | 74.1 | 61.6 | 59.8 | 97.3 | 79.5 | 62.5 |  |

Upper diagonal values are for nucleotide, lower diagonal values correspond to amino acids

| *Rpl2* | **Subtype 1 NandII** | **Subtype 2 Flemming** | **Subtype 3 DMP/08-326** | **Subtype 3 DMP/ 08-1043** | **Subtype 3 IH:478** | **Subtype 4 DMP/02-328** | **Subtype 4 DMP/10-212** | **Subtype 6 SSI 754** | **Subtype 7 S7-B** | **Subtype 8 DMP/08-128** | **Subtype 9 F5323** |
| --- | --- | --- | --- | --- | --- | --- | --- | --- | --- | --- | --- |
| **Subtype 1 NandII** |  | 86.3 | 80.3 | 80.3 | 80.2 | 74.2 | 74.6 | 78.8 | 80.7 | 74.3 | 78.8 |
| **Subtype 2 Flemming** | 88 |  | 78.8 | 78.8 | 78.7 | 74.3 | 74.8 | 79.8 | 79.7 | 73.5 | 79.3 |
| **Subtype 3 DMP/08-326** | 77.2 | 77.2 |  | 95.4 | 99.6 | 74.6 | 75 | 76.7 | 77.4 | 77 | 76.7 |
| **Subtype 3DMP/ 08-1043** | 77.6 | 76.8 | 95.2 |  | 95.6 | 74.6 | 74.6 | 76.8 | 77 | 76.6 | 76.4 |
| **Subtype 3 IH:478** | 76.8 | 77.2 | 98.8 | 95.6 |  | 74.4 | 75 | 76.8 | 77.5 | 76.8 | 76.8 |
| **Subtype 4 DMP/02-328** | 69.6 | 70.4 | 68.8 | 70.4 | 68.8 |  | 86.7 | 74.3 | 74.8 | 76.8 | 74 |
| **Subtype 4 DMP/10-212** | 68.8 | 71.2 | 68.8 | 69.2 | 69.2 | 87.2 |  | 74.2 | 77 | 78.2 | 74 |
| **Subtype 6 SSI 754** | 76.4 | 73.6 | 70.4 | 71.2 | 70.8 | 66.4 | 67.2 |  | 82.6 | 71.8 | 95.6 |
| **Subtype 7 S7-B** | 76.4 | 73.6 | 72.4 | 72.8 | 72.8 | 69.2 | 70 | 77.2 |  | 73.1 | 82.5 |
| **Subtype 8 DMP/08-128** | 70.8 | 71.2 | 72.8 | 72.8 | 72.4 | 74.8 | 76 | 66.8 | 67.6 |  | 71.6 |
| **Subtype 9 F5323** | 76 | 73.6 | 70.4 | 70.8 | 70.8 | 65.6 | 67.6 | 95.6 | 77.6 | 66 |  |

Upper diagonal values are for nucleotide, lower diagonal values correspond to amino acids

| *Rps19* | **Subtype 1 NandII** | **Subtype 2 Flemming** | **Subtype 3 DMP/08-326** | **Subtype 3 DMP/ 08-1043** | **Subtype 3 IH:478** | **Subtype 4 DMP/02-328** | **Subtype 4 DMP/10-212** | **Subtype 6 SSI 754** | **Subtype 7 S7-B** | **Subtype 8 DMP/08-128** | **Subtype 9 F5323** |
| --- | --- | --- | --- | --- | --- | --- | --- | --- | --- | --- | --- |
| **Subtype 1 NandII** |  | 84.9 | 80 | 79.1 | 80 | 70.7 | 75.1 | 76 | 80 | 72.4 | 76 |
| **Subtype 2 Flemming** | 76 |  | 81.3 | 82.2 | 81.3 | 71.6 | 77.3 | 77.8 | 79.1 | 78.7 | 79.6 |
| **Subtype 3 DMP/08-326** | 69.3 | 66.7 |  | 98.2 | 100 | 74.2 | 77.3 | 78.7 | 78.2 | 78.2 | 78.7 |
| **Subtype 3DMP/ 08-1043** | 66.7 | 68 | 97.3 |  | 98.2 | 73.8 | 76.9 | 77.8 | 76.9 | 77.8 | 78.7 |
| **Subtype 3 IH:478** | 69.3 | 66.7 | 100 | 97.3 |  | 74.2 | 77.3 | 78.7 | 78.2 | 78.2 | 78.7 |
| **Subtype 4 DMP/02-328** | 60 | 60 | 61.3 | 60 | 61.3 |  | 87.6 | 68.4 | 69.8 | 77.3 | 68.9 |
| **Subtype 4 DMP/10-212** | 58.7 | 64 | 58.7 | 58.7 | 58.7 | 82.7 |  | 75.1 | 76 | 82.7 | 76.4 |
| **Subtype 6 SSI 754** | 60 | 61.3 | 61.3 | 60 | 61.3 | 50.7 | 82.7 |  | 85.8 | 73.3 | 97.3 |
| **Subtype 7 S7-B** | 60 | 61.3 | 58.7 | 56 | 58.7 | 52 | 50.7 | 80 |  | 74.2 | 86.7 |
| **Subtype 8 DMP/08-128** | 62.7 | 70.7 | 68 | 66.7 | 68 | 64 | 52 | 57.3 | 57.3 |  | 73.8 |
| **Subtype 9 F5323** | 60 | 64 | 61.3 | 62.7 | 61.3 | 52 | 64 | 97.3 | 80 | 56 |  |

Upper diagonal values are for nucleotide, lower diagonal values correspond to amino acids

| *Rps3* | **Subtype 1 NandII** | **Subtype 2 Flemming** | **Subtype 3 DMP/08-326** | **Subtype 3 DMP/ 08-1043** | **Subtype 3 IH:478** | **Subtype 4 DMP/02-328** | **Subtype 4 DMP/10-212** | **Subtype 6 SSI 754** | **Subtype 7 S7-B** | **Subtype 8 DMP/08-128** | **Subtype 9 F5323** |
| --- | --- | --- | --- | --- | --- | --- | --- | --- | --- | --- | --- |
| **Subtype 1 NandII** |  | 86.6 | 76.5 | 75.8 | 76.1 | 73.1 | 73.1 | 81.2 | 79.8 | 72.2 | 81.3 |
| **Subtype 2 Flemming** | 80.1 |  | 75.1 | 73.5 | 74.7 | 70.6 | 70.9 | 78.5 | 77.9 | 71.1 | 79.3 |
| **Subtype 3 DMP/08-326** | 61.1 | 61.5 |  | 96 | 99.6 | 72.4 | 72.1 | 74.4 | 73.8 | 71.8 | 74.8 |
| **Subtype 3DMP/ 08-1043** | 60.8 | 60.1 | 95.3 |  | 96 | 71.1 | 70.7 | 73.6 | 73.1 | 70.5 | 73.9 |
| **Subtype 3 IH:478** | 61.1 | 61.5 | 99.3 | 95.3 |  | 72 | 72.1 | 74.1 | 73.5 | 71.6 | 74.6 |
| **Subtype 4 DMP/02-328** | 55.7 | 52.4 | 54.4 | 53.7 | 54.4 |  | 86.6 | 70 | 70 | 76.2 | 71.1 |
| **Subtype 4 DMP/10-212** | 57.4 | 53 | 53.7 | 54.1 | 53.7 | 78 |  | 70.5 | 70.8 | 74.6 | 70.9 |
| **Subtype 6 SSI 754** | 66.2 | 64.9 | 57.8 | 57.1 | 57.4 | 51 | 51.4 |  | 84.7 | 71.9 | 96.7 |
| **Subtype 7 S7-B** | 64.2 | 64.5 | 55.4 | 54.7 | 55.4 | 48.3 | 50 | 73.6 |  | 70.5 | 85.3 |
| **Subtype 8 DMP/08-128** | 55.4 | 54.1 | 51 | 50 | 51 | 63.5 | 62.2 | 49.7 | 51.4 |  | 72.4 |
| **Subtype 9 F5323** | 66.2 | 65.2 | 57.4 | 56.8 | 57.1 | 52.4 | 52 | 94.3 | 73.6 | 50.3 |  |

Upper diagonal values are for nucleotide, lower diagonal values correspond to amino acids

| *Rpl16* | **Subtype 1 NandII** | **Subtype 2 Flemming** | **Subtype 3 DMP/08-326** | **Subtype 3 DMP/ 08-1043** | **Subtype 3 IH:478** | **Subtype 4 DMP/02-328** | **Subtype 4 DMP/10-212** | **Subtype 6 SSI 754** | **Subtype 7 S7-B** | **Subtype 8 DMP/08-128** | **Subtype 9 F5323** |
| --- | --- | --- | --- | --- | --- | --- | --- | --- | --- | --- | --- |
| **Subtype 1 NandII** |  | 90.5 | 82.7 | 82.2 | 82.7 | 74.7 | 77.6 | 85.2 | 82.5 | 75.2 | 85.4 |
| **Subtype 2 Flemming** | 93.4 |  | 82.5 | 82 | 82.5 | 73.7 | 74.2 | 83.7 | 80 | 74.5 | 83 |
| **Subtype 3 DMP/08-326** | 80.9 | 78.7 |  | 95.1 | 100 | 78.8 | 81 | 87.1 | 84.4 | 79.8 | 86.1 |
| **Subtype 3DMP/ 08-1043** | 80.9 | 79.4 | 99.3 |  | 95.1 | 78.6 | 79.8 | 87.1 | 84.2 | 80.3 | 85.6 |
| **Subtype 3 IH:478** | 80.9 | 78.7 | 100 | 99.3 |  | 78.8 | 81 | 87.1 | 84.4 | 79.8 | 86.1 |
| **Subtype 4 DMP/02-328** | 72.1 | 67.6 | 76.5 | 76.5 | 76.5 |  | 90.5 | 80.3 | 79.6 | 81.3 | 79.3 |
| **Subtype 4 DMP/10-212** | 72.8 | 69.9 | 78.7 | 78.7 | 78.7 | 76.5 |  | 80.5 | 80 | 83.5 | 80.3 |
| **Subtype 6 SSI 754** | 77.2 | 75 | 86 | 86 | 86 | 78.7 | 73.5 |  | 86.4 | 79.8 | 97.6 |
| **Subtype 7 S7-B** | 78.7 | 77.2 | 86.8 | 86 | 86.8 | 86 | 75.7 | 86 |  | 78.3 | 86.6 |
| **Subtype 8 DMP/08-128** | 69.1 | 69.1 | 75.7 | 75.7 | 75.7 | 86.8 | 83.8 | 71.3 | 72.1 |  | 78.8 |
| **Subtype 9 F5323** | 77.9 | 75.7 | 83.8 | 83.8 | 83.8 | 75.7 | 71.3 | 96.3 | 84.6 | 69.9 |  |

Upper diagonal values are for nucleotide, lower diagonal values correspond to amino acids.

| *nad7* | **Subtype 1 NandII** | **Subtype 2 Flemming** | **Subtype 3 DMP/08-326** | **Subtype 3 DMP/ 08-1043** | **Subtype 3 IH:478** | **Subtype 4 DMP/02-328** | **Subtype 4 DMP/10-212** | **Subtype 6 SSI 754** | **Subtype 7 S7-B** | **Subtype 8 DMP/08-128** | **Subtype 9 F5323** |
| --- | --- | --- | --- | --- | --- | --- | --- | --- | --- | --- | --- |
| **Subtype 1 NandII** |  | 94.8 | 92.4 | 92 | 92.3 | 88.5 | 88.2 | 92 | 93.7 | 87.2 | 92.3 |
| **Subtype 2 Flemming** | 98.2 |  | 90.7 | 90.2 | 90.6 | 88.7 | 87.8 | 91.5 | 92 | 87 | 91.8 |
| **Subtype 3 DMP/08-326** | 92.4 | 92.4 |  | 98.3 | 99.9 | 90.1 | 90.1 | 92.1 | 92.9 | 88.2 | 92 |
| **Subtype 3DMP/ 08-1043** | 92.4 | 92.4 | 100 |  | 98.2 | 89.9 | 89.9 | 91.8 | 92.4 | 87.8 | 91.9 |
| **Subtype 3 IH:478** | 92.4 | 92.4 | 100 | 100 |  | 89.9 | 89.9 | 92 | 92.8 | 88.1 | 91.9 |
| **Subtype 4 DMP/02-328** | 92.4 | 92.4 | 95.1 | 95.1 | 95.1 |  | 94.4 | 88.7 | 88.7 | 91.8 | 89 |
| **Subtype 4 DMP/10-212** | 92.9 | 92.4 | 95.1 | 95.1 | 95.1 | 98.2 |  | 88.2 | 88.7 | 91.8 | 88.2 |
| **Subtype 6 SSI 754** | 17.8 | 17.8 | 17.8 | 17.8 | 17.8 | 18.2 | 18.7 |  | 93.1 | 88.2 | 98.8 |
| **Subtype 7 S7-B** | 91.6 | 92 | 93.8 | 93.8 | 93.8 | 93.3 | 92.9 | 16.9 |  | 87.4 | 93.3 |
| **Subtype 8 DMP/08-128** | 92.4 | 92.4 | 94.7 | 94.7 | 94.7 | 97.8 | 97.8 | 18.7 | 92.4 |  | 87.8 |
| **Subtype 9 F5323** | 93.3 | 93.3 | 94.7 | 94.7 | 94.7 | 93.8 | 93.8 | 19.1 | 96.9 | 93.3 |  |

Upper diagonal values are for nucleotide, lower diagonal values correspond to amino acids

| *Orf160* | **Subtype 1 NandII** | **Subtype 2 Flemming** | **Subtype 3 DMP/08-326** | **Subtype 3 DMP/ 08-1043** | **Subtype 3 IH:478** | **Subtype 4 DMP/02-328** | **Subtype 4 DMP/10-212** | **Subtype 6 SSI 754** | **Subtype 7 S7-B** | **Subtype 8 DMP/08-128** | **Subtype 9 F5323** |
| --- | --- | --- | --- | --- | --- | --- | --- | --- | --- | --- | --- |
| **Subtype 1 NandII** |  | 75.9 | 69.1 | 67.9 | 69.1 | 63.5 | 62 | 68.9 | 73.5 | 62 | 72.3 |
| **Subtype 2 Flemming** | 64.1 |  | 70.1 | 70.3 | 70.1 | 63 | 61.6 | 71 | 74.5 | 61.6 | 71.3 |
| **Subtype 3 DMP/08-326** | 46.5 | 52.1 |  | 93.9 | 100 | 65.7 | 64 | 68.9 | 70.6 | 63 | 69.3 |
| **Subtype 3DMP/ 08-1043** | 45.1 | 50.7 | 93 |  | 93.9 | 65.2 | 64.5 | 67.6 | 69.1 | 63.5 | 68.4 |
| **Subtype 3 IH:478** | 46.5 | 52.1 | 100 | 93 |  | 65.7 | 64 | 68.9 | 70.6 | 63 | 69.3 |
| **Subtype 4 DMP/02-328** | 32.4 | 30.3 | 33.1 | 33.1 | 33.1 |  | 80 | 61.3 | 63.7 | 64.7 | 62.3 |
| **Subtype 4 DMP/10-212** | 34.5 | 35.9 | 41.5 | 40.8 | 41.5 | 63.4 |  | 60.6 | 59.9 | 63.5 | 60.8 |
| **Subtype 6 SSI 754** | 45.1 | 48.6 | 41.5 | 43.7 | 41.5 | 33.1 | 35.2 |  | 73.7 | 60.6 | 93.9 |
| **Subtype 7 S7-B** | 51.4 | 52.1 | 45.1 | 44.4 | 45.1 | 31.7 | 33.8 | 49.3 |  | 60.8 | 74.9 |
| **Subtype 8 DMP/08-128** | 28.9 | 32.4 | 37.3 | 37.3 | 37.3 | 40.8 | 45.1 | 27.5 | 28.2 |  | 60.8 |
| **Subtype 9 F5323** | 45.1 | 47.9 | 43 | 44.4 | 43 | 35.2 | 35.2 | 90.1 | 49.3 | 29.6 |  |

Upper diagonal values are for nucleotide, lower diagonal values correspond to amino acids

| *Nad4* | **Subtype 1 NandII** | **Subtype 2 Flemming** | **Subtype 3 DMP/08-326** | **Subtype 3 DMP/ 08-1043** | **Subtype 3 IH:478** | **Subtype 4 DMP/02-328** | **Subtype 4 DMP/10-212** | **Subtype 6 SSI 754** | **Subtype 7 S7-B** | **Subtype 8 DMP/08-128** | **Subtype 9 F5323** |
| --- | --- | --- | --- | --- | --- | --- | --- | --- | --- | --- | --- |
| **Subtype 1 NandII** |  | 92.4 | 90.4 | 90.8 | 90.4 | 86.5 | 87 | 88.9 | 89.5 | 86.6 | 88.4 |
| **Subtype 2 Flemming** | 96.7 |  | 91.2 | 91.5 | 91.3 | 86.7 | 87.4 | 89.1 | 89.1 | 85.9 | 88.4 |
| **Subtype 3 DMP/08-326** | 93 | 94 |  | 97 | 99.9 | 87.8 | 88.4 | 89.5 | 89.8 | 86.8 | 89.3 |
| **Subtype 3DMP/ 08-1043** | 93 | 94 | 99.6 |  | 97.1 | 88 | 89.1 | 89.5 | 89.8 | 87.8 | 89.6 |
| **Subtype 3 IH:478** | 93.2 | 94.3 | 99.8 | 99.8 |  | 87.8 | 88.4 | 89.5 | 90 | 86.8 | 89.4 |
| **Subtype 4 DMP/02-328** | 88.9 | 89.9 | 90.6 | 90.8 | 90.8 |  | 93.4 | 86.5 | 85.1 | 88.4 | 86.1 |
| **Subtype 4 DMP/10-212** | 89.3 | 90.3 | 91 | 91.4 | 91.2 | 99 |  | 87.3 | 85.2 | 88 | 87.3 |
| **Subtype 6 SSI 754** | 91.6 | 91.8 | 91.8 | 92.2 | 92 | 88.5 | 89.3 |  | 90 | 86.2 | 97.9 |
| **Subtype 7 S7-B** | 92.4 | 92.2 | 92 | 92 | 92.2 | 86.7 | 87.3 | 93 |  | 83.8 | 90.3 |
| **Subtype 8 DMP/08-128** | 91.4 | 91.4 | 92.6 | 92.8 | 92.8 | 92.2 | 92.6 | 89.9 | 88.9 |  | 86.4 |
| **Subtype 9 F5323** | 91.6 | 91.8 | 92.2 | 92.6 | 92.4 | 88.9 | 89.7 | 99.6 | 93 | 90.3 |  |

Upper diagonal values are for nucleotide, lower diagonal values correspond to amino acids

| *Rps4* | **Subtype 1 NandII** | **Subtype 2 Flemming** | **Subtype 3 DMP/08-326** | **Subtype 3 DMP/ 08-1043** | **Subtype 3 IH:478** | **Subtype 4 DMP/02-328** | **Subtype 4 DMP/10-212** | **Subtype 6 SSI 754** | **Subtype 7 S7-B** | **Subtype 8 DMP/08-128** | **Subtype 9 F5323** |
| --- | --- | --- | --- | --- | --- | --- | --- | --- | --- | --- | --- |
| **Subtype 1 NandII** |  | 80.9 | 69.8 | 69.5 | 69.9 | 65.8 | 65.3 | 72.4 | 71.1 | 64.7 | 72.5 |
| **Subtype 2 Flemming** | 69.3 |  | 69.3 | 69 | 69.4 | 66.1 | 64.5 | 71.6 | 69.8 | 64 | 71.6 |
| **Subtype 3 DMP/08-326** | 40.4 | 42.3 |  | 92.7 | 99.9 | 67.9 | 67.5 | 69.1 | 67.4 | 65.8 | 69.3 |
| **Subtype 3DMP/ 08-1043** | 41.4 | 43.2 | 88.7 |  | 92.8 | 67.4 | 67.3 | 69.8 | 67.6 | 66.2 | 69.8 |
| **Subtype 3 IH:478** | 40.6 | 42.5 | 99.8 | 89 |  | 68.1 | 67.7 | 69.2 | 67.6 | 65.8 | 69.4 |
| **Subtype 4 DMP/02-328** | 35.9 | 38.2 | 37.4 | 37.2 | 37.5 |  | 78 | 65 | 65 | 68.9 | 65.5 |
| **Subtype 4 DMP/10-212** | 34.3 | 36.2 | 36.3 | 37.2 | 36.4 | 62.6 |  | 64.7 | 63.6 | 68.2 | 64.8 |
| **Subtype 6 SSI 754** | 44.2 | 45.9 | 39.4 | 39.8 | 39.5 | 34.9 | 33.7 |  | 76.7 | 63.4 | 94.1 |
| **Subtype 7 S7-B** | 44.6 | 45.4 | 39.3 | 40.2 | 39.4 | 35.8 | 33.1 | 54.6 |  | 62.5 | 77.2 |
| **Subtype 8 DMP/08-128** | 36.6 | 36.6 | 37.5 | 38.2 | 37.5 | 45 | 45.6 | 33.4 | 34.6 |  | 63.6 |
| **Subtype 9 F5323** | 44.8 | 46.9 | 39.9 | 40.6 | 40 | 34.6 | 34.3 | 87.4 | 54 | 34.1 |  |

Upper diagonal values are for nucleotide, lower diagonal values correspond to amino acids

| *Rps14* | **Subtype 1 NandII** | **Subtype 2 Flemming** | **Subtype 3 DMP/08-326** | **Subtype 3 DMP/ 08-1043** | **Subtype 3 IH:478** | **Subtype 4 DMP/02-328** | **Subtype 4 DMP/10-212** | **Subtype 6 SSI 754** | **Subtype 7 S7-B** | **Subtype 8 DMP/08-128** | **Subtype 9 F5323** |
| --- | --- | --- | --- | --- | --- | --- | --- | --- | --- | --- | --- |
| **Subtype 1 NandII** |  | 87.3 | 77.6 | 77.3 | 77.6 | 75.5 | 75.8 | 83.9 | 82.4 | 78.8 | 83.6 |
| **Subtype 2 Flemming** | 79.8 |  | 76.7 | 75.8 | 76.7 | 73 | 75.5 | 82.7 | 83.6 | 75.5 | 83.3 |
| **Subtype 3 DMP/08-326** | 67.9 | 64.2 |  | 94.5 | 100 | 71.5 | 72.4 | 73.6 | 75.8 | 77 | 74.2 |
| **Subtype 3DMP/ 08-1043** | 67.9 | 63.3 | 95.4 |  | 94.5 | 70.9 | 72.1 | 72.1 | 75.8 | 76.4 | 72.7 |
| **Subtype 3 IH:478** | 67.9 | 64.2 | 100 | 95.4 |  | 71.5 | 72.4 | 73.6 | 75.8 | 77 | 74.2 |
| **Subtype 4 DMP/02-328** | 59.6 | 58.7 | 58.7 | 56.9 | 58.7 |  | 86.4 | 74.2 | 72.1 | 77 | 73.3 |
| **Subtype 4 DMP/10-212** | 60.6 | 59.6 | 61.5 | 59.6 | 61.5 | 80.7 |  | 75.2 | 74.8 | 77 | 74.5 |
| **Subtype 6 SSI 754** | 75.2 | 71.6 | 61.5 | 60.6 | 61.5 | 58.7 | 63.3 |  | 83.6 | 74.5 | 95.5 |
| **Subtype 7 S7-B** | 70.6 | 70.6 | 64.2 | 62.4 | 64.2 | 56 | 60.6 | 76.1 |  | 77.3 | 83 |
| **Subtype 8 DMP/08-128** | 67 | 64.2 | 69.7 | 67.9 | 69.7 | 62.4 | 64.2 | 65.1 | 67 |  | 75.2 |
| **Subtype 9 F5323** | 74.3 | 71.6 | 59.6 | 58.7 | 59.6 | 58.7 | 60.6 | 92.7 | 75.2 | 66.1 |  |

Upper diagonal values are for nucleotide, lower diagonal values correspond to amino acids

| *Rps8* | **Subtype 1 NandII** | **Subtype 2 Flemming** | **Subtype 3 DMP/08-326** | **Subtype 3 DMP/ 08-1043** | **Subtype 3 IH:478** | **Subtype 4 DMP/02-328** | **Subtype 4 DMP/10-212** | **Subtype 6 SSI 754** | **Subtype 7 S7-B** | **Subtype 8 DMP/08-128** | **Subtype 9 F5323** |
| --- | --- | --- | --- | --- | --- | --- | --- | --- | --- | --- | --- |
| **Subtype 1 NandII** |  | 79.6 | 73.7 | 73.5 | 73.7 | 59 | 63.5 | 74.5 | 75.1 | 60.6 | 74.8 |
| **Subtype 2 Flemming** | 69.5 |  | 70.2 | 70.2 | 70.2 | 58.4 | 63 | 73.5 | 71.3 | 59 | 73.5 |
| **Subtype 3 DMP/08-326** | 59.4 | 56.3 |  | 95.7 | 99.7 | 65.7 | 66.2 | 74.5 | 73.2 | 66.5 | 75.1 |
| **Subtype 3DMP/ 08-1043** | 60.2 | 57 | 96.9 |  | 96 | 66.2 | 66 | 74.5 | 73.7 | 66 | 75.1 |
| **Subtype 3 IH:478** | 59.4 | 56.3 | 99.2 | 97.7 |  | 66 | 66.5 | 74.5 | 73.2 | 66.8 | 75.1 |
| **Subtype 4 DMP/02-328** | 46.1 | 40.6 | 51.6 | 51.6 | 51.6 |  | 81.2 | 64.6 | 63.5 | 68.4 | 65.1 |
| **Subtype 4 DMP/10-212** | 43.8 | 42.2 | 49.2 | 50.8 | 49.2 | 73.4 |  | 64.6 | 65.7 | 69.2 | 65.1 |
| **Subtype 6 SSI 754** | 57 | 55.5 | 64.1 | 64.8 | 64.1 | 46.1 | 48.4 |  | 84.5 | 64.1 | 97.3 |
| **Subtype 7 S7-B** | 60.2 | 57.8 | 62.5 | 61.7 | 62.5 | 46.9 | 49.2 | 78.9 |  | 63.5 | 85 |
| **Subtype 8 DMP/08-128** | 43 | 39.8 | 49.2 | 48.4 | 49.2 | 53.1 | 50.8 | 45.3 | 46.1 |  | 64.1 |
| **Subtype 9 F5323** | 57 | 57 | 66.4 | 67.2 | 66.4 | 46.9 | 49.2 | 94.5 | 80.5 | 45.3 |  |

Upper diagonal values are for nucleotide, lower diagonal values correspond to amino acids

| *Rpl6* | **Subtype 1 NandII** | **Subtype 2 Flemming** | **Subtype 3 DMP/08-326** | **Subtype 3 DMP/ 08-1043** | **Subtype 3 IH:478** | **Subtype 4 DMP/02-328** | **Subtype 4 DMP/10-212** | **Subtype 6 SSI 754** | **Subtype 7 S7-B** | **Subtype 8 DMP/08-128** | **Subtype 9 F5323** |
| --- | --- | --- | --- | --- | --- | --- | --- | --- | --- | --- | --- |
| **Subtype 1 NandII** |  | 85.7 | 75.1 | 77.6 | 75.1 | 73.7 | 71.6 | 77.8 | 80.2 | 71 | 77.6 |
| **Subtype 2 Flemming** | 77.8 |  | 74.7 | 76.3 | 74.7 | 72.4 | 69.6 | 79.2 | 78 | 70.4 | 79 |
| **Subtype 3 DMP/08-326** | 61.4 | 59.1 |  | 94.3 | 100 | 73.9 | 69.4 | 73.9 | 73.7 | 69.6 | 73.7 |
| **Subtype 3DMP/ 08-1043** | 61.4 | 57.9 | 95.3 |  | 94.3 | 75.3 | 70.2 | 73.3 | 74.1 | 70.4 | 72.4 |
| **Subtype 3 IH:478** | 61.4 | 59.1 | 100 | 95.3 |  | 73.9 | 69.4 | 73.9 | 73.7 | 69.6 | 73.7 |
| **Subtype 4 DMP/02-328** | 55.6 | 51.5 | 57.3 | 56.7 | 57.3 |  | 83.1 | 73.1 | 72.4 | 73.7 | 73.5 |
| **Subtype 4 DMP/10-212** | 55.6 | 55 | 55 | 54.4 | 55 | 74.9 |  | 69.6 | 71.4 | 71.2 | 69.2 |
| **Subtype 6 SSI 754** | 65.5 | 66.7 | 59.6 | 57.9 | 59.6 | 53.2 | 52.6 |  | 79.6 | 72.2 | 95.7 |
| **Subtype 7 S7-B** | 68.4 | 65.5 | 60.8 | 59.6 | 60.8 | 51.5 | 53.2 | 70.2 |  | 69.6 | 80 |
| **Subtype 8 DMP/08-128** | 53.8 | 48 | 53.8 | 53.2 | 53.8 | 56.7 | 55.6 | 50.3 | 50.3 |  | 72 |
| **Subtype 9 F5323** | 66.7 | 67.3 | 60.2 | 58.5 | 60.2 | 52.6 | 52 | 94.2 | 69.6 | 50.9 |  |

Upper diagonal values are for nucleotide, lower diagonal values correspond to amino acids

| *Orf192 (Rps2)* | **Subtype 1 NandII** | **Subtype 2 Flemming** | **Subtype 3 DMP/08-326** | **Subtype 3 DMP/ 08-1043** | **Subtype 3 IH:478** | **Subtype 4 DMP/02-328** | **Subtype 4 DMP/10-212** | **Subtype 6 SSI 754** | **Subtype 7 S7-B** | **Subtype 8 DMP/08-128** | **Subtype 9 F5323** |
| --- | --- | --- | --- | --- | --- | --- | --- | --- | --- | --- | --- |
| **Subtype 1 NandII** |  | 82.2 | 73.7 | 73.5 | 73.7 | 67.3 | 67.3 | 72.6 | 72.6 | 69 | 73.3 |
| **Subtype 2 Flemming** | 76 |  | 72.4 | 73 | 72.4 | 69.5 | 68.4 | 71.7 | 72.4 | 68.8 | 72.8 |
| **Subtype 3 DMP/08-326** | 62.8 | 62.3 |  | 95.8 | 99.6 | 64.8 | 67.2 | 70.1 | 73.1 | 71 | 70.6 |
| **Subtype 3DMP/ 08-1043** | 62.3 | 63.4 | 96.2 |  | 95.8 | 65 | 66.8 | 70.2 | 73.3 | 70.2 | 70.6 |
| **Subtype 3 IH:478** | 62.8 | 62.3 | 98.9 | 96.2 |  | 64.8 | 80 | 64.1 | 63.9 | 68.8 | 63.7 |
| **Subtype 4 DMP/02-328** | 54.1 | 57.9 | 51.4 | 51.9 | 51.4 |  | 80 | 64.1 | 63.9 | 68.8 | 63.7 |
| **Subtype 4 DMP/10-212** | 51.9 | 54.1 | 51.4 | 50.8 | 50.3 | 68.9 |  | 62.3 | 61.7 | 65.9 | 62.4 |
| **Subtype 6 SSI 754** | 56.3 | 54.6 | 54.1 | 54.6 | 54.1 | 47.5 | 42.6 |  | 78 | 64.8 | 96 |
| **Subtype 7 S7-B** | 54.1 | 57.4 | 54.1 | 55.2 | 54.1 | 47 | 43.2 | 65.6 |  | 65.9 | 78.9 |
| **Subtype 8 DMP/08-128** | 55.7 | 59.6 | 59.6 | 59 | 59.6 | 61.2 | 54.1 | 50.8 | 48.6 |  | 64.2 |
| **Subtype 9 F5323** | 57.4 | 55.7 | 54.1 | 54.6 | 54.1 | 47.5 | 43.2 | 94.5 | 66.7 | 50.9 |  |

Upper diagonal values are for nucleotide, lower diagonal values correspond to amino acids

| *Rpl14* | **Subtype 1 NandII** | **Subtype 2 Flemming** | **Subtype 3 DMP/08-326** | **Subtype 3 DMP/ 08-1043** | **Subtype 3 IH:478** | **Subtype 4 DMP/02-328** | **Subtype 4 DMP/10-212** | **Subtype 6 SSI 754** | **Subtype 7 S7-B** | **Subtype 8 DMP/08-128** | **Subtype 9 F5323** |
| --- | --- | --- | --- | --- | --- | --- | --- | --- | --- | --- | --- |
| **Subtype 1 NandII** |  | 86.9 | 82.3 | 82.1 | 82.8 | 74 | 74.7 | 83.1 | 81.1 | 80.8 | 81.8 |
| **Subtype 2 Flemming** | 88.5 |  | 80.6 | 82.1 | 81.1 | 76.3 | 77.5 | 82.1 | 79.8 | 81.1 | 81.8 |
| **Subtype 3 DMP/08-326** | 78.6 | 77.1 |  | 95.2 | 99.5 | 75.8 | 75.3 | 78.8 | 79.3 | 80.6 | 79.5 |
| **Subtype 3DMP/ 08-1043** | 79.4 | 77.9 | 95.4 |  | 95.7 | 75.3 | 75.8 | 79 | 80.3 | 80.3 | 80.1 |
| **Subtype 3 IH:478** | 78.6 | 77.1 | 99.2 | 96.2 |  | 76.3 | 75.8 | 79.3 | 79.8 | 81.1 | 80.1 |
| **Subtype 4 DMP/02-328** | 66.4 | 65.6 | 67.2 | 67.2 | 67.9 |  | 89.4 | 73 | 75.5 | 81.8 | 73.2 |
| **Subtype 4 DMP/10-212** | 67.9 | 67.9 | 67.2 | 68.7 | 67.9 | 90.8 |  | 75 | 75 | 81.1 | 74.7 |
| **Subtype 6 SSI 754** | 74 | 74.8 | 72.5 | 74 | 73.3 | 60.3 | 62.6 |  | 83.8 | 79.8 | 98 |
| **Subtype 7 S7-B** | 74 | 73.3 | 71.8 | 74 | 72.5 | 67.9 | 65.6 | 80.2 |  | 79.5 | 84.3 |
| **Subtype 8 DMP/08-128** | 72.5 | 73.3 | 74.8 | 77.1 | 75.6 | 82.4 | 78.6 | 67.9 | 75.6 |  | 50.3 |
| **Subtype 9 F5323** | 73.3 | 74 | 73.3 | 74.8 | 74 | 60.3 | 62.6 | 99.2 | 79.4 | 67.9 |  |

Upper diagonal values are for nucleotide, lower diagonal values correspond to amino acids

| *Orf175 (rpl5)* | **Subtype 1 NandII** | **Subtype 2 Flemming** | **Subtype 3 DMP/08-326** | **Subtype 3 DMP/ 08-1043** | **Subtype 3 IH:478** | **Subtype 4 DMP/02-328** | **Subtype 4 DMP/10-212** | **Subtype 6 SSI 754** | **Subtype 7 S7-B** | **Subtype 8 DMP/08-128** | **Subtype 9 F5323** |
| --- | --- | --- | --- | --- | --- | --- | --- | --- | --- | --- | --- |
| **Subtype 1 NandII** |  | 78.9 | 68.2 | 70.3 | 68.4 | 64 | 64.8 | 74.3 | 72.8 | 66.7 | 73.5 |
| **Subtype 2 Flemming** | 67.2 |  | 71.4 | 72.8 | 71.6 | 65.1 | 62.5 | 73.5 | 74.7 | 64.8 | 74.5 |
| **Subtype 3 DMP/08-326** | 54.6 | 56.3 |  | 95.4 | 99.4 | 65.9 | 62.3 | 70.1 | 69 | 64.6 | 69.5 |
| **Subtype 3DMP/ 08-1043** | 54.6 | 56.3 | 96 |  | 95.6 | 67 | 64.6 | 71.8 | 70.9 | 65.5 | 71.4 |
| **Subtype 3 IH:478** | 54.6 | 56.9 | 99.4 | 96.6 |  | 66.1 | 62.5 | 70.3 | 69.1 | 65 | 69.7 |
| **Subtype 4 DMP/02-328** | 40.2 | 45.4 | 44.8 | 44.3 | 44.8 |  | 81.3 | 64.6 | 64.2 | 68.8 | 64.6 |
| **Subtype 4 DMP/10-212** | 42.5 | 41.4 | 42.5 | 42 | 42.5 | 70.7 |  | 63.6 | 62.3 | 66.1 | 63.8 |
| **Subtype 6 SSI 754** | 60.9 | 61.5 | 49.4 | 50.6 | 50 | 42.5 | 43.7 |  | 83.2 | 65.9 | 95.8 |
| **Subtype 7 S7-B** | 58 | 62.6 | 48.3 | 49.4 | 48.9 | 42 | 40.2 | 74.1 |  | 66.5 | 83.4 |
| **Subtype 8 DMP/08-128** | 46 | 47.7 | 44.8 | 45.4 | 45.4 | 50 | 48.3 | 46 | 46 |  | 66.5 |
| **Subtype 9 F5323** | 59.2 | 60.9 | 48.9 | 50 | 49.4 | 42.5 | 43.7 | 94.8 | 73.6 | 44.8 |  |

Upper diagonal values are for nucleotide, lower diagonal values correspond to amino acids

| *nad 5* | **Subtype 1 NandII** | **Subtype 2 Flemming** | **Subtype 3 DMP/08-326** | **Subtype 3 DMP/ 08-1043** | **Subtype 3 IH:478** | **Subtype 4 DMP/02-328** | **Subtype 4 DMP/10-212** | **Subtype 6 SSI 754** | **Subtype 7 S7-B** | **Subtype 8 DMP/08-128** | **Subtype 9 F5323** |
| --- | --- | --- | --- | --- | --- | --- | --- | --- | --- | --- | --- |
| **Subtype 1 NandII** |  | 91.8 | 86.2 | 85.4 | 86.1 | 81.9 | 82.8 | 85.9 | 86.9 | 79.9 | 85.6 |
| **Subtype 2 Flemming** |  |  | 86.8 | 86.3 | 86.7 | 82.7 | 84.2 | 86.4 | 87.1 | 81.2 | 85.8 |
| **Subtype 3 DMP/08-326** |  |  |  | 96 | 99.9 | 85 | 86 | 85.2 | 86.9 | 81.9 | 84.5 |
| **Subtype 3DMP/ 08-1043** |  |  |  |  | 95.9 | 84.8 | 85.6 | 84.3 | 86.6 | 81.8 | 83.8 |
| **Subtype 3 IH:478** |  |  |  |  |  | 84.9 | 85.9 | 85.1 | 86.8 | 81.8 | 84.4 |
| **Subtype 4 DMP/02-328** |  |  |  |  |  |  | 90.2 | 81.4 | 82.5 | 82.9 | 81.2 |
| **Subtype 4 DMP/10-212** |  |  |  |  |  |  |  | 82.8 | 84.2 | 84.3 | 82.2 |
| **Subtype 6 SSI 754** |  |  |  |  |  |  |  |  | 90.2 | 79.9 | 97.6 |
| **Subtype 7 S7-B** |  |  |  |  |  |  |  |  |  | 79.4 | 89.4 |
| **Subtype 8 DMP/08-128** |  |  |  |  |  |  |  |  |  |  | 79.9 |
| **Subtype 9 F5323** |  |  |  |  |  |  |  |  |  |  |  |

Upper diagonal values are for nucleotide, lower diagonal values correspond to amino acids

| *Rps11* | **Subtype 1 NandII** | **Subtype 2 Flemming** | **Subtype 3 DMP/08-326** | **Subtype 3 DMP/ 08-1043** | **Subtype 3 IH:478** | **Subtype 4 DMP/02-328** | **Subtype 4 DMP/10-212** | **Subtype 6 SSI 754** | **Subtype 7 S7-B** | **Subtype 8 DMP/08-128** | **Subtype 9 F5323** |
| --- | --- | --- | --- | --- | --- | --- | --- | --- | --- | --- | --- |
| **Subtype 1 NandII** |  | 83.3 | 69.5 | 69.3 | 69.5 | 65.9 | 65.9 | 66.7 | 69.7 | 67.4 | 65.7 |
| **Subtype 2 Flemming** | 79.2 |  | 68.9 | 68.7 | 68.9 | 65 | 65 | 69.1 | 67 | 67 | 68 |
| **Subtype 3 DMP/08-326** | 51.6 | 50.3 |  | 95.3 | 100 | 72.3 | 71.7 | 67.6 | 68 | 70.2 | 66.1 |
| **Subtype 3DMP/ 08-1043** | 50.9 | 49.7 | 97.5 |  | 95.3 | 72.5 | 71.9 | 66.3 | 66.1 | 70.8 | 65.2 |
| **Subtype 3 IH:478** | 48.4 | 47.2 | 95.6 | 93.1 |  | 72.3 | 71.7 | 67.6 | 68 | 70.2 | 66.1 |
| **Subtype 4 DMP/02-328** | 47.2 | 50.3 | 59.1 | 58.5 | 56 |  | 82.8 | 65.9 | 65.2 | 71 | 66.1 |
| **Subtype 4 DMP/10-212** | 45.3 | 45.3 | 54.1 | 54.1 | 50.9 | 75.5 |  | 64.4 | 64.8 | 71.7 | 64.8 |
| **Subtype 6 SSI 754** | 42.8 | 44.7 | 47.2 | 45.9 | 44.7 | 44.7 | 39 |  | 80.3 | 65 | 95.1 |
| **Subtype 7 S7-B** | 45.9 | 45.3 | 45.3 | 44 | 42.8 | 45.3 | 39.6 | 68.6 |  | 62.9 | 79.6 |
| **Subtype 8 DMP/08-128** | 47.2 | 49.1 | 50.3 | 50.9 | 46.5 | 56.6 | 54.7 | 39.6 | 43.4 |  | 63.5 |
| **Subtype 9 F5323** | 41.5 | 43.4 | 46.5 | 45.9 | 44 | 44.7 | 38.4 | 97.5 | 69.2 | 40.3 |  |

Upper diagonal values are for nucleotide, lower diagonal values correspond to amino acids

| *Nad 9* | **Subtype 1 NandII** | **Subtype 2 Flemming** | **Subtype 3 DMP/08-326** | **Subtype 3 DMP/ 08-1043** | **Subtype 3 IH:478** | **Subtype 4 DMP/02-328** | **Subtype 4 DMP/10-212** | **Subtype 6 SSI 754** | **Subtype 7 S7-B** | **Subtype 8 DMP/08-128** | **Subtype 9 F5323** |
| --- | --- | --- | --- | --- | --- | --- | --- | --- | --- | --- | --- |
| **Subtype 1 NandII** |  | 93.7 | 84.9 | 85.1 | 84.9 | 80.9 | 81 | 80.7 | 86 | 80.4 | 81.4 |
| **Subtype 2 Flemming** | 97.4 |  | 85.3 | 85.1 | 85.3 | 82.7 | 82.9 | 81.6 | 85.8 | 81.4 | 82.4 |
| **Subtype 3 DMP/08-326** | 82.7 | 83.7 |  | 98.5 | 100 | 85.4 | 84.3 | 83.2 | 85.3 | 83.1 | 83.6 |
| **Subtype 3DMP/ 08-1043** | 82.1 | 83.2 | 99.5 |  | 98.5 | 85.8 | 85.1 | 83.1 | 85.1 | 83.9 | 83.1 |
| **Subtype 3 IH:478** | 82.7 | 83.7 | 100 | 99.5 |  | 85.4 | 84.3 | 83.2 | 85.3 | 83.1 | 83.6 |
| **Subtype 4 DMP/02-328** | 78.6 | 79.6 | 87.2 | 86.7 | 87.2 |  | 91.7 | 77.8 | 80.2 | 84.6 | 78.7 |
| **Subtype 4 DMP/10-212** | 80.6 | 81.6 | 89.3 | 88.8 | 89.3 | 95.9 |  | 76.8 | 79.4 | 87.1 | 78 |
| **Subtype 6 SSI 754** | 81.1 | 82.1 | 84.7 | 84.2 | 84.7 | 77 | 79.6 |  | 87.1 | 77.5 | 97 |
| **Subtype 7 S7-B** | 86.2 | 85.2 | 85.7 | 85.2 | 85.7 | 77.6 | 79.6 | 87.8 |  | 80.7 | 87.8 |
| **Subtype 8 DMP/08-128** | 81.1 | 82.1 | 88.3 | 87.8 | 88.3 | 90.3 | 91.3 | 80.6 | 82.1 |  | 78.7 |
| **Subtype 9 F5323** | 81.1 | 82.1 | 85.7 | 85.2 | 85.7 | 78.1 | 80.6 | 99 | 87.8 | 81.6 |  |

Upper diagonal values are for nucleotide, lower diagonal values correspond to amino acids

| *Rps10* | **Subtype 1 NandII** | **Subtype 2 Flemming** | **Subtype 3 DMP/08-326** | **Subtype 3 DMP/ 08-1043** | **Subtype 3 IH:478** | **Subtype 4 DMP/02-328** | **Subtype 4 DMP/10-212** | **Subtype 6 SSI 754** | **Subtype 7 S7-B** | **Subtype 8 DMP/08-128** | **Subtype 9 F5323** |
| --- | --- | --- | --- | --- | --- | --- | --- | --- | --- | --- | --- |
| **Subtype 1 NandII** |  | 86.9 | 66.9 | 66.9 | 66.9 | 64.7 | 66.9 | 72.8 | 76.3 | 66.9 | 71.9 |
| **Subtype 2 Flemming** | 78.1 |  | 70.6 | 71.9 | 70.6 | 65.6 | 67.8 | 75.6 | 76.9 | 70.6 | 75 |
| **Subtype 3 DMP/08-326** | 45.7 | 47.6 |  | 92.8 | 99.4 | 64.4 | 65 | 65.3 | 65.6 | 64.7 | 64.4 |
| **Subtype 3DMP/ 08-1043** | 44.8 | 49.5 | 94.3 |  | 92.8 | 63.8 | 64.1 | 65.6 | 66.3 | 65.3 | 64.7 |
| **Subtype 3 IH:478** | 45.7 | 48.6 | 98.1 | 94.3 |  | 65 | 65.6 | 65.3 | 65.6 | 64.7 | 64.1 |
| **Subtype 4 DMP/02-328** | 48.6 | 51.4 | 46.7 | 46.7 | 47.6 |  | 79.1 | 63.8 | 68.8 | 71.9 | 62.8 |
| **Subtype 4 DMP/10-212** | 45.7 | 47.6 | 49.5 | 49.5 | 51.4 | 71.4 |  | 62.8 | 68.1 | 72.8 | 62.8 |
| **Subtype 6 SSI 754** | 55.2 | 56.2 | 42.9 | 43.8 | 43.8 | 45.7 | 45.7 |  | 87.8 | 70.3 | 97.2 |
| **Subtype 7 S7-B** | 60 | 61.9 | 45.7 | 46.7 | 46.7 | 50.5 | 53.3 | 78.1 |  | 73.4 | 86.6 |
| **Subtype 8 DMP/08-128** | 53.3 | 56.2 | 46.7 | 48.6 | 47.6 | 61.9 | 57.1 | 55.2 | 61 |  | 68.8 |
| **Subtype 9 F5323** | 55.2 | 55.2 | 43.8 | 43.8 | 43.8 | 44.8 | 44.8 | 97.1 | 77.1 | 53.3 |  |

Upper diagonal values are for nucleotide, lower diagonal values correspond to amino acids

| *nad 2* | **Subtype 1 NandII** | **Subtype 2 Flemming** | **Subtype 3 DMP/08-326** | **Subtype 3 DMP/ 08-1043** | **Subtype 3 IH:478** | **Subtype 4 DMP/02-328** | **Subtype 4 DMP/10-212** | **Subtype 6 SSI 754** | **Subtype 7 S7-B** | **Subtype 8 DMP/08-128** | **Subtype 9 F5323** |
| --- | --- | --- | --- | --- | --- | --- | --- | --- | --- | --- | --- |
| **Subtype 1 NandII** |  | 91 | 86.3 | 86.5 | 86.3 | 83.5 | 83.2 | 85.2 | 86.4 | 81.4 | 85.5 |
| **Subtype 2 Flemming** | 94.5 |  | 86.6 | 86.6 | 86.6 | 82.9 | 83.7 | 83.9 | 86.5 | 81.2 | 84.3 |
| **Subtype 3 DMP/08-326** | 89.5 | 88.6 |  | 97.3 | 99.9 | 82.5 | 82.5 | 84.5 | 85.6 | 82.3 | 85 |
| **Subtype 3DMP/ 08-1043** | 89.5 | 88.6 | 99.6 |  | 97.2 | 82.8 | 83.1 | 85.1 | 85.9 | 81.9 | 85.4 |
| **Subtype 3 IH:478** | 89.2 | 88.4 | 99.8 | 99.4 |  | 82.5 | 82.5 | 84.4 | 85.5 | 82.3 | 84.8 |
| **Subtype 4 DMP/02-328** | 83.4 | 81.9 | 82.8 | 82.8 | 82.6 |  | 92.3 | 81.9 | 82.8 | 84.7 | 82.9 |
| **Subtype 4 DMP/10-212** | 83.6 | 82.2 | 82.4 | 82.4 | 82.2 | 95.5 |  | 81.9 | 82.5 | 84.5 | 82.8 |
| **Subtype 6 SSI 754** | 85.8 | 85.4 | 86.2 | 86.2 | 86 | 82.2 | 80.5 |  | 85.6 | 80 | 97.9 |
| **Subtype 7 S7-B** | 87.8 | 87.4 | 86.8 | 86.8 | 86.6 | 82.4 | 81.9 | 88.4 |  | 79.9 | 86.1 |
| **Subtype 8 DMP/08-128** | 82.4 | 81.9 | 81.5 | 81.5 | 81.3 | 86.2 | 85.6 | 80.7 | 80.5 |  | 80.6 |
| **Subtype 9 F5323** | 86 | 85.6 | 86.4 | 86.4 | 86.2 | 82.8 | 81.1 | 99.2 | 88.2 | 80.7 |  |

Upper diagonal values are for nucleotide, lower diagonal values correspond to amino acids

| *nad 11* | **Subtype 1 NandII** | **Subtype 2 Flemming** | **Subtype 3 DMP/08-326** | **Subtype 3 DMP/ 08-1043** | **Subtype 3 IH:478** | **Subtype 4 DMP/02-328** | **Subtype 4 DMP/10-212** | **Subtype 6 SSI 754** | **Subtype 7 S7-B** | **Subtype 8 DMP/08-128** | **Subtype 9 F5323** |
| --- | --- | --- | --- | --- | --- | --- | --- | --- | --- | --- | --- |
| **Subtype 1 NandII** |  | 89.3 | 81.3 | 81.5 | 81.3 | 79.5 | 78.3 | 81.3 | 82.7 | 77.4 | 81.4 |
| **Subtype 2 Flemming** | 85.8 |  | 82.3 | 82.8 | 82.3 | 78.7 | 78.1 | 82.3 | 83.3 | 78.1 | 82.2 |
| **Subtype 3 DMP/08-326** | 75.8 | 78 |  | 97.1 | 99.9 | 80.1 | 79.3 | 78.4 | 79.2 | 79 | 78.3 |
| **Subtype 3DMP/ 08-1043** | 76.2 | 78.1 | 99 |  | 97.1 | 80.6 | 79.8 | 78.5 | 79.9 | 79.2 | 78.4 |
| **Subtype 3 IH:478** | 75.9 | 78.1 | 99.9 | 99.1 |  | 80.1 | 79.3 | 78.4 | 79.2 | 79 | 78.3 |
| **Subtype 4 DMP/02-328** | 73 | 72.2 | 74.3 | 74.6 | 74.3 |  | 88 | 74.8 | 75.6 | 82.6 | 75.3 |
| **Subtype 4 DMP/10-212** | 71.2 | 71.4 | 73.3 | 73.4 | 73.3 | 89.3 |  | 74.8 | 75.9 | 81.6 | 75.4 |
| **Subtype 6 SSI 754** | 74 | 74.3 | 70.9 | 71.4 | 71.1 | 66.4 | 66.8 |  | 85.2 | 74.8 | 97.3 |
| **Subtype 7 S7-B** | 75.9 | 76.8 | 72.5 | 73 | 72.7 | 67.3 | 67 | 82.1 |  | 74.7 | 84.8 |
| **Subtype 8 DMP/08-128** | 69.2 | 69.5 | 73.4 | 73.3 | 73.4 | 78 | 76.9 | 64.6 | 64.2 |  | 75.2 |
| **Subtype 9 F5323** | 74.6 | 74.4 | 70.6 | 71.1 | 70.8 | 66.8 | 67 | 98.2 | 82.2 | 64.9 |  |

Upper diagonal values are for nucleotide, lower diagonal values correspond to amino acids

| *nad 1* | **Subtype 1 NandII** | **Subtype 2 Flemming** | **Subtype 3 DMP/08-326** | **Subtype 3 DMP/ 08-1043** | **Subtype 3 IH:478** | **Subtype 4 DMP/02-328** | **Subtype 4 DMP/10-212** | **Subtype 6 SSI 754** | **Subtype 7 S7-B** | **Subtype 8 DMP/08-128** | **Subtype 9 F5323** |
| --- | --- | --- | --- | --- | --- | --- | --- | --- | --- | --- | --- |
| **Subtype 1 NandII** |  | 92.4 | 89.7 | 89.6 | 89.7 | 85.3 | 85.1 | 88.7 | 89.3 | 84.1 | 88.3 |
| **Subtype 2 Flemming** | 96 |  | 88.3 | 87.9 | 88.3 | 84.7 | 83.9 | 87.5 | 87.6 | 84.3 | 87.3 |
| **Subtype 3 DMP/08-326** | 89.6 | 89 |  | 97.7 | 100 | 87.5 | 87.2 | 88.7 | 88.9 | 86.7 | 88.8 |
| **Subtype 3DMP/ 08-1043** | 89.3 | 88.7 | 99.4 |  | 97.7 | 87.9 | 87.3 | 89.2 | 89.2 | 87.2 | 88.9 |
| **Subtype 3 IH:478** | 89.6 | 89 | 100 | 99.4 |  | 87.5 | 87.2 | 88.7 | 88.9 | 86.7 | 88.8 |
| **Subtype 4 DMP/02-328** | 86.3 | 86 | 92.1 | 92.1 | 92.1 |  | 92 | 87 | 87.5 | 87.7 | 87.4 |
| **Subtype 4 DMP/10-212** | 87.2 | 86.3 | 91.8 | 92.1 | 91.8 | 97.6 |  | 86.5 | 87.3 | 88.8 | 87.2 |
| **Subtype 6 SSI 754** | 89.3 | 88.7 | 91.5 | 91.5 | 91.5 | 90.5 | 90.9 |  | 91.4 | 85 | 98.7 |
| **Subtype 7 S7-B** | 89 | 89 | 89.9 | 89.9 | 89.9 | 90.2 | 90.5 | 92.4 |  | 85.3 | 91.1 |
| **Subtype 8 DMP/08-128** | 87.8 | 87.2 | 93 | 93 | 93 | 93.6 | 95.1 | 90.2 | 90.2 |  | 85.4 |
| **Subtype 9 F5323** | 89 | 88.4 | 91.8 | 91.8 | 91.8 | 90.2 | 90.5 | 99.7 | 92.1 | 90.5 |  |

Upper diagonal values are for nucleotide, lower diagonal values correspond to amino acids

| *nad 4L* | **Subtype 1 NandII** | **Subtype 2 Flemming** | **Subtype 3 DMP/08-326** | **Subtype 3 DMP/ 08-1043** | **Subtype 3 IH:478** | **Subtype 4 DMP/02-328** | **Subtype 4 DMP/10-212** | **Subtype 6 SSI 754** | **Subtype 7 S7-B** | **Subtype 8 DMP/08-128** | **Subtype 9 F5323** |
| --- | --- | --- | --- | --- | --- | --- | --- | --- | --- | --- | --- |
| **Subtype 1 NandII** |  | 91.7 | 87.1 | 86.8 | 87.1 | 81.5 | 82.8 | 84.2 | 85.8 | 80.9 | 84.2 |
| **Subtype 2 Flemming** | 98 |  | 88.4 | 89.4 | 88.4 | 83.5 | 85.1 | 86.8 | 88.4 | 84.2 | 86.5 |
| **Subtype 3 DMP/08-326** | 94 | 94 |  | 98.3 | 100 | 87.5 | 88.8 | 86.8 | 86.5 | 87.5 | 86.1 |
| **Subtype 3DMP/ 08-1043** | 94 | 94 | 100 |  | 98.3 | 87.8 | 89.1 | 87.1 | 87.1 | 88.1 | 86.1 |
| **Subtype 3 IH:478** | 94 | 94 | 100 | 100 |  | 87.5 | 88.8 | 86.8 | 86.5 | 87.5 | 86.1 |
| **Subtype 4 DMP/02-328** | 92 | 92 | 96 | 96 | 96 |  | 91.1 | 86.1 | 85.1 | 91.1 | 85.1 |
| **Subtype 4 DMP/10-212** | 93 | 93 | 97 | 97 | 97 | 97 |  | 85.1 | 84.8 | 89.1 | 84.5 |
| **Subtype 6 SSI 754** | 91 | 91 | 90 | 90 | 90 | 90 | 89 |  | 92.4 | 85.5 | 99 |
| **Subtype 7 S7-B** | 91 | 91 | 89 | 89 | 89 | 90 | 88 | 96 |  | 85.5 | 91.4 |
| **Subtype 8 DMP/08-128** | 91 | 91 | 95 | 95 | 95 | 96 | 95 | 88 | 88 |  | 84.5 |
| **Subtype 9 F5323** | 91 | 91 | 90 | 90 | 90 | 90 | 89 | 100 | 96 | 88 |  |

Upper diagonal values are for nucleotide, lower diagonal values correspond to amino acids

| *Orf143 (Rps7)* | **Subtype 1 NandII** | **Subtype 2 Flemming** | **Subtype 3 DMP/08-326** | **Subtype 3 DMP/ 08-1043** | **Subtype 3 IH:478** | **Subtype 4 DMP/02-328** | **Subtype 4 DMP/10-212** | **Subtype 6 SSI 754** | **Subtype 7 S7-B** | **Subtype 8 DMP/08-128** | **Subtype 9 F5323** |
| --- | --- | --- | --- | --- | --- | --- | --- | --- | --- | --- | --- |
| **Subtype 1 NandII** |  | 87.9 | 77.5 | 79.6 | 77.5 | 76.8 | 75.9 | 74.7 | 76.1 | 78 | 74.9 |
| **Subtype 2 Flemming** | 88.1 |  | 80.7 | 81.7 | 80.7 | 76.3 | 77 | 75.4 | 76.1 | 78.2 | 74.9 |
| **Subtype 3 DMP/08-326** | 70.6 | 72.7 |  | 97.7 | 100 | 78.7 | 77.7 | 75.6 | 75.6 | 80.3 | 75.2 |
| **Subtype 3DMP/ 08-1043** | 69.2 | 71.3 | 97.9 |  | 97.7 | 80 | 78.7 | 75.4 | 76.6 | 81.2 | 75.4 |
| **Subtype 3 IH:478** | 69.2 | 71.3 | 97.9 | 100 |  | 78.7 | 77.7 | 75.6 | 75.6 | 80.3 | 75.2 |
| **Subtype 4 DMP/02-328** | 66.4 | 68.5 | 69.2 | 69.2 | 69.2 |  | 90.7 | 72.2 | 71.9 | 85.2 | 72.6 |
| **Subtype 4 DMP/10-212** | 67.1 | 69.9 | 69.9 | 69.9 | 69.9 | 88.1 |  | 71 | 69.8 | 86.1 | 71 |
| **Subtype 6 SSI 754** | 57.3 | 57.3 | 58.7 | 58.7 | 58.7 | 54.5 | 54.5 |  | 77 | 74 | 98.6 |
| **Subtype 7 S7-B** | 61.5 | 60.8 | 60.8 | 60.1 | 60.1 | 53.8 | 53.1 | 61.5 |  | 72.4 | 76.1 |
| **Subtype 8 DMP/08-128** | 67.8 | 69.9 | 74.8 | 74.8 | 74.8 | 81.1 | 80.4 | 53.8 | 55.2 |  | 74 |
| **Subtype 9 F5323** | 56.6 | 56.6 | 58.7 | 58.7 | 58.7 | 55.2 | 54.5 | 97.9 | 60.1 | 54.5 |  |

Upper diagonal values are for nucleotide, lower diagonal values correspond to amino acids

| *Rps12* | **Subtype 1 NandII** | **Subtype 2 Flemming** | **Subtype 3 DMP/08-326** | **Subtype 3 DMP/ 08-1043** | **Subtype 3 IH:478** | **Subtype 4 DMP/02-328** | **Subtype 4 DMP/10-212** | **Subtype 6 SSI 754** | **Subtype 7 S7-B** | **Subtype 8 DMP/08-128** | **Subtype 9 F5323** |
| --- | --- | --- | --- | --- | --- | --- | --- | --- | --- | --- | --- |
| **Subtype 1 NandII** |  | 86.2 | 84.3 | 85.1 | 84 | 77.7 | 78.7 | 82.7 | 85.4 | 79.3 | 83 |
| **Subtype 2 Flemming** | 85.6 |  | 80.9 | 81.1 | 80.6 | 78.2 | 81.1 | 82.2 | 82.7 | 79.5 | 82.4 |
| **Subtype 3 DMP/08-326** | 82.4 | 80 |  | 98.4 | 99.7 | 79.5 | 81.9 | 80.9 | 82.7 | 79.5 | 80.6 |
| **Subtype 3DMP/ 08-1043** | 81.6 | 80 | 97.6 |  | 98.1 | 79.5 | 82.4 | 81.1 | 83.8 | 79.8 | 81.1 |
| **Subtype 3 IH:478** | 81.6 | 80 | 98.4 | 99.2 |  | 79.3 | 81.6 | 80.9 | 82.7 | 79.5 | 80.6 |
| **Subtype 4 DMP/02-328** | 80 | 80 | 80 | 79.2 | 79.2 |  | 90.2 | 79.5 | 77.1 | 81.6 | 79.5 |
| **Subtype 4 DMP/10-212** | 80.8 | 81.6 | 82.4 | 81.6 | 81.6 | 91.2 |  | 80.9 | 79.5 | 84.6 | 81.1 |
| **Subtype 6 SSI 754** | 80.8 | 82.4 | 80.8 | 81.6 | 81.6 | 80.8 | 83.2 |  | 87.5 | 80.1 | 98.1 |
| **Subtype 7 S7-B** | 86.4 | 84.8 | 81.6 | 81.6 | 81.6 | 78.4 | 80.8 | 88.8 |  | 80.1 | 88 |
| **Subtype 8 DMP/08-128** | 84.8 | 85.6 | 80 | 80.8 | 80.8 | 83.2 | 85.6 | 81.6 | 80.8 |  | 79.8 |
| **Subtype 9 F5323** | 82.4 | 82.4 | 82.4 | 81.6 | 81.6 | 83.2 | 85.6 | 97.6 | 88.8 | 80.8 |  |

Upper diagonal values are for nucleotide, lower diagonal values correspond to amino acids
